# Health outcomes and cost-effectiveness of diversion programs for low-level drug offenders: A model-based analysis

**DOI:** 10.1371/journal.pmed.1003239

**Published:** 2020-10-13

**Authors:** Cora L. Bernard, Isabelle J. Rao, Konner K. Robison, Margaret L. Brandeau

**Affiliations:** Department of Management Science and Engineering, Stanford University, Stanford, California, United States of America; Massachusetts General Hospital, UNITED STATES

## Abstract

**Background:**

Cycles of incarceration, drug abuse, and poverty undermine ongoing public health efforts to reduce overdose deaths and the spread of infectious disease in vulnerable populations. Jail diversion programs aim to divert low-level drug offenders toward community care resources, avoiding criminal justice costs and disruptions in treatment for HIV, hepatitis C virus (HCV), and drug abuse. We sought to assess the health benefits and cost-effectiveness of a jail diversion program for low-level drug offenders.

**Methods and findings:**

We developed a microsimulation model, calibrated to King County, Washington, that captured the spread of HIV and HCV infections and incarceration and treatment systems as well as preexisting interventions such as needle and syringe programs and opiate agonist therapy. We considered an adult population of people who inject drugs (PWID), people who use drugs but do not inject (PWUD), men who have sex with men, and lower-risk heterosexuals. We projected discounted lifetime costs and quality-adjusted life years (QALYs) over a 10-year time horizon with and without a jail diversion program and calculated resulting incremental cost-effectiveness ratios (ICERs) from the health system and societal perspectives. We also tracked HIV and HCV infections, overdose deaths, and jail population size.

Over 10 years, the program was estimated to reduce HIV and HCV incidence by 3.4% (95% CI 2.7%–4.0%) and 3.3% (95% CI 3.1%–3.4%), respectively, overdose deaths among PWID by 10.0% (95% CI 9.8%–10.8%), and jail population size by 6.3% (95% CI 5.9%–6.7%). When considering healthcare costs only, the program cost $25,500/QALY gained (95% CI $12,600–$48,600). Including savings from reduced incarceration (societal perspective) improved the ICER to $6,200/QALY gained (95% CI, cost-saving $24,300). Sensitivity analysis indicated that cost-effectiveness depends on diversion program participants accessing community programs such as needle and syringe programs, treatment for substance use disorder, and HIV and HCV treatment, as well as diversion program cost.

A limitation of the analysis is data availability, as fewer data are available for diversion programs than for more established interventions aimed at people with substance use disorder. Additionally, like any model of a complex system, our model relies on simplifying assumptions: For example, we simplified pathways in the healthcare and criminal justice systems, modeled an average efficacy for substance use disorder treatment, and did not include costs associated with homelessness, unemployment, and breakdown in family structure.

**Conclusions:**

We found that diversion programs for low-level drug offenders are likely to be cost-effective, generating savings in the criminal justice system while only moderately increasing healthcare costs. Such programs can reduce incarceration and its associated costs, and also avert overdose deaths and improve quality of life for PWID, PWUD, and the broader population (through reduced HIV and HCV transmission).

## Abstract 2

Cora Bernard and co-workers investigate the health and societal value of diversion programs for people who misuse drugs.

## Author summary

**Why was this study done?:**

- Strategies for people with substance use disorder based on criminalization and aggressive law enforcement have created barriers to harm reduction while having little or no impact on the number of people who use drugs.
- Diversion programs redirect individuals with low-level drug and prostitution offenses away from the criminal justice system and into community-based services such as treatment for substance use disorder, housing, and mental health care.
- Our model-based analysis was designed to assess the health benefits and cost-effectiveness of a jail diversion program for low-level drug offenders.

**What did the researchers do and find?:**

- We developed a stochastic microsimulation model of adults in King County, Washington that captures sexual and injecting-based risk behaviors and partnerships and tracks the 10-year trajectories of people who inject drugs, people who use drugs but do not inject, and the lower-risk general population, with and without a diversion program.
- We projected HIV and hepatitis C virus (HCV) incidence, fatal overdoses, jail population size, and lifetime costs and quality-adjusted life years (QALYs) experienced in the population, with and without a diversion program.
- We estimated that the diversion program would reduce cumulative HIV and HCV incidence, fatal overdoses, and the jailed population, would lead to gains in QALYs, and would be highly cost-effective.

**What do these findings mean?:**

- A 2016 United Nations AIDS Programme (UNAIDS) report found that the status quo of criminalizing drug abuse is failing from the public safety and public health perspectives. Our study demonstrates that these perspectives can be aligned.
- Our analysis suggests that diversion programs are likely to substantially reduce spending in the criminal justice system while moderately increasing spending in the healthcare sector and are likely to be highly cost-effective.
- The spread of infectious disease stemming from drug injection and the global overcrowding of jails and prisons suggest that, more than ever, effective, cost-effective, and innovative approaches are required to address these crises. Diversion programs are one such program.

## Introduction

A 2016 United Nations AIDS Programme (UNAIDS) report found that “strategies [for people who inject drugs (PWID)] based on criminalization and aggressive law enforcement have created barriers to harm reduction while having little or no impact on the number of people who use drugs” [1]. Cycles of incarceration, drug abuse, and poverty undermine ongoing public health efforts to reduce overdose deaths and the spread of infectious disease in vulnerable populations [2,3]. Jails are incarceration facilities for those awaiting trial or held for minor crimes, and prisons are for individuals convicted of serious crimes. Individuals at high risk of detainment in jails such as PWID and people who use illicit drugs (other than marijuana) but do not inject them (PWUD) are critical agents in the spread of HIV and hepatitis C virus (HCV) [4]. Moreover, poor health resources within jails and lack of post-release services interrupt care for individuals receiving treatment for substance use disorder, HIV, or HCV [5–7], and the abrupt withdrawal from drugs while in jail increases the likelihood of overdose upon release [8–12]. Interrupting these mechanisms of incarceration and transmission is imperative in order to control HIV and HCV and to reduce morbidity and mortality among PWID and PWUD populations globally.

Diversion programs include drug courts, which divert individuals to treatment for substance use disorder with intensive supervision and regular court appearances, and jail diversion programs, which bypass the criminal justice system and focus on a broader array of community services. Drug courts have been shown to reduce state and county government costs [13], as well as recidivism [14, 15], but typically miss the opportunity to address healthcare issues [16]. Jail diversion programs such as Law Enforcement Assisted Diversion (LEAD), a pilot program in King County, Washington (the Seattle metropolitan area), redirect individuals with low-level drug and prostitution offenses away from the criminal justice system and into community-based services such as treatment for substance use disorder, housing, and mental health care [17,18]. Consistent with LEAD’s stated goal “to improve public safety and public order” [19], LEAD has demonstrated effectiveness in decreasing recidivism rates for its clients: An average participant is 58% less likely to be rearrested and saves the county $8,000 in legal fees and processing costs annually [20–22]. The substantiated links between incarceration and public health suggest that reducing jail populations and increasing the rate at which drug offenders enter sustained treatment for substance use disorder is likely to generate additional benefits. A financial analysis of the LEAD program, which did not consider healthcare-related costs, found it to be cost-saving [19,20]. However, such a program may increase total healthcare costs, offsetting savings in criminal justice costs.

To quantify these effects and evaluate the effectiveness and cost-effectiveness of a jail diversion program from a public health perspective, we developed a stochastic microsimulation model of adults in King County, Washington. The model captured sexual and injecting-based risk behaviors and partnerships and tracks the 10-year trajectories of PWID, PWUD, and the lower-risk general population, with and without a diversion program. We calculated HIV and HCV infections averted, overdose deaths averted, and change in incarcerated population due to the diversion program. We calculated lifetime discounted costs and quality-adjusted life years (QALYs) from a health system perspective as well as from a societal perspective that includes criminal justice system costs, and we calculated resulting incremental cost-effectiveness ratios (ICERs) for the diversion program compared to no intervention.

## Methods

### Design

**Fig 1:**
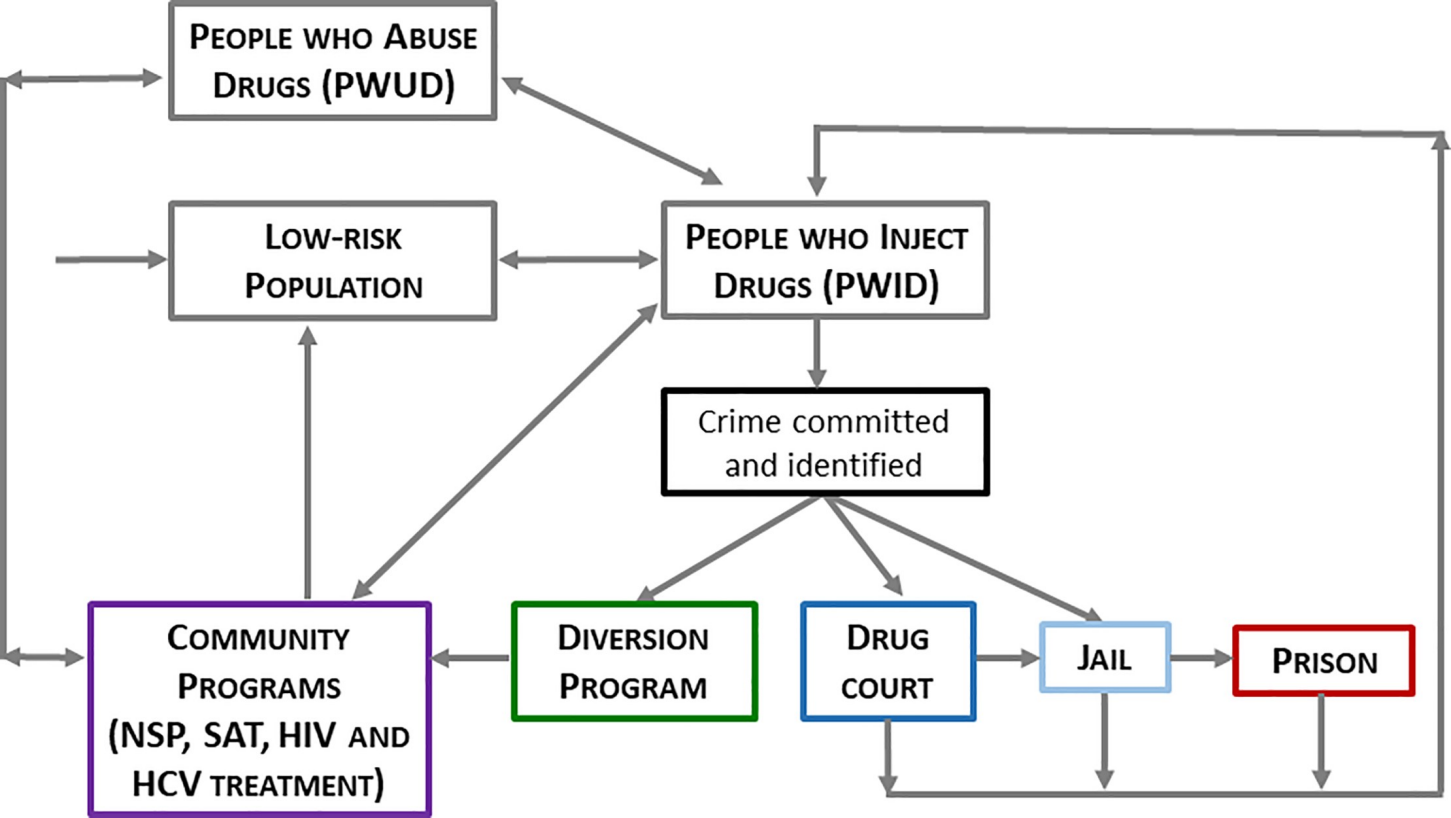
Schematic of jail diversion model. PWUD and PWID flow in and out of community programs, via which a small percentage move to lower-risk populations. In the absence of a diversion program, individuals move to jail at the time a crime is committed and identified and will possibly move on to prison, depending on the nature of the crime. A diversion program diverts individuals from the criminal justice system into community programs. In this simplified model schematic, some arrows (e.g., deaths) have been omitted for visual clarity. HCV, hepatitis C virus; NSP, needle and syringe program; PWID, people who inject drugs; PWUD, individuals with substance use disorder who do not inject drugs; SUDT, treatment for substance use disorder.

Our model (Fig 1) stratified the adult, King County population by age (18–19, 20–29, 30–39, 40–49, 50–59, or 60–74), sex, sexual orientation (men who have sex with men [MSM], heterosexual male, heterosexual female), risk group (PWID, PWUD, or low-risk), race (Black, white, or other), HIV and HCV status, disease awareness status, disease treatment status, drug treatment status, and incarceration status (unincarcerated, jail, or prison). Individuals formed injecting or sexual partnerships and moved between treatment and incarceration statuses with probabilities specific to their demographic groups. We programmed the model in Python and calibrated it to King County epidemiologic targets. Table 1 provides an overview of key model inputs. S1 Text provides the Consolidated Health Economic Evaluation Reporting System (CHEERS) checklist, and S2 Text provides full model details.

**Table 1:** Overview of key model inputs.

Input	Description	Table(s) Showing Values
Model demographics	Urban population reflective of King County, Washington, stratified by age, sex, sexual orientation, race, HIV and HCV status, drug use disorder status, drug treatment status, and incarceration status	Table 2; Tables A, B, C, E, F in S2 Text
Risk behavior	Monthly needlesharing and sexual partnership formation	Table D in S2 Text
	Drug injection, sexual contact, and condom use behavior	Table H in S2 Text
Population transitions	Incarceration transitions: rates of identified crime, transitions to prison or jail, release from prison	Table G in S2 Text
	HIV and HCV transmission via sexual and injecting contacts	Table I in S2 Text
	HIV and HCV progression	Tables J and K in S2 Text
	HIV and HCV awareness and treatment	Table L in S2 Text
	Entry into and exit from substance use disorder treatment SUDT and NSPs	Table M in S2 Text
	Change in drug use status (e.g., transition from PWUD to PWID, or quitting drug use)	Table O in S2 Text
	Deaths due to background mortality rate, HIV or HCV infection, and drug overdose	Table P in S2 Text
Costs	Costs of background healthcare, HIV and HCV infection, SUDT, NSPs, and incarceration	Table Q in S2 Text
QALYs	QALY multipliers for males and females by age, by drug use status, HIV and HCV status, and incarceration status	Table Q in S2 Text
Diversion program	Reduction in criminal activity and incarceration, change in QALY value, changes in rates of joining and quitting community programs (SUDT, NSPs, and HIV and HCV treatment), and annual program costs per person	Table N in S2 Text

HCV, hepatitis C virus; NSP, needle and syringe program; PWID, people who inject drugs; PWUD, individuals with substance use disorder who do not inject drugs; QALY, quality-adjusted life year, SUDT, treatment for substance use disorder.

### Population

In King County, 1.7% of the adult population are PWID, 6% are PWUD, and the rest are considered low risk (Table 2) [23–27]. MSM and people of color comprise 2.6% and 30% of the total population, respectively, but make up approximately 10% and 43%, respectively, of the drug-misusing populations [23–26]. HIV prevalence is highest among individuals who are both MSM and PWID [23–26], HCV prevalence is highest among PWID [24,28–30], and incarceration rates are highest among Black male PWID [31–34]. Individuals in the simulated population were assigned demographic characteristics at the start of the model run.

**Table 2:** Key model demographics.

	PWID (0.017)														
By Sexual Orientation	Heterosexual Female (0.326)					Heterosexual Male (0.579)					Men Who Have Sex with Men (0.095)				
**By age**	**18–29** (0.151)	**30–39** (0.249)	**40–49** (0.267)	**50–59** (0.250)	**60–74** (0.083)	**18–29** (0.153)	**30–39** (0.248)	**40–49** (0.263)	**50–59** (0.252)	**60–74** (0.084)	**18–29** (0.126)	**30–39** (0.268)	**40–49** (0.323)	**50–59** (0.212)	**60–74** (0.071)
**HIV**	0.038	0.070	0.087	0.041	0.041	0.019	0.035	0.044	0.020	0.020	0.385	0.541	0.599	0.399	0.399
**HCV**	0.450	0.533	0.637	0.783	0.783	0.450	0.533	0.637	0.783	0.783	0.450	0.533	0.637	0.783	0.783
**Incarceration**	0.083	0.043	0.026	0.015	0.016	0.297	0.149	0.091	0.055	0.058	0.297	0.149	0.091	0.055	0.058
**By race**	**Black**		**Other**	**White**		**Black**		**Other**	**White**		**Black**		**Other**	**White**	
**HIV**	0.071		0.076	0.050		0.080		0.043	0.004		0.500		0.500	0.500	
**HCV**	0.631		0.631	0.632		0.631		0.631	0.631		0.631		0.631	0.631	
**Incarceration**	0.051		0.015	0.036		0.175		0.052	0.130		0.175		0.052	0.130	

Parenthetical values correspond to the percent that the current row’s subpopulation makes up of the next highest (sub)population: For example, PWID make up 1.7% of the model population, and 32.6% of PWID are heterosexual female. HIV, HCV, and incarceration rows refer to the demographic prevalence within each subpopulation.

HCV, hepatitis C virus; HIV, human immunodeficiency virus; PWID, people who inject drugs.

### Simulation

We simulated 140,000 individuals in each model run (approximately 10% of King County’s population). The model ran over a 10-year time horizon in weekly time steps. To simulate behavioral and health events, we associated a set of transition probabilities with each individual based on demographic data. Every location (e.g., unincarcerated, in jail, in prison), disease status, and risk status in the model was associated with a healthcare cost and, possibly, criminal justice-specific cost as well as a QALY value. An individual’s weekly costs and QALYs were calculated as, respectively, a sum or multiple of all states characterizing that individual at that time. For each scenario (with the diversion program and without), we ran the model 2,500 times. We calculated average values for each outcome over the 2,500 runs as well as the fifth and 95th percentile value.

### Measure: Incarceration

In the model, an individual could commit a misdemeanor or felony [31]. Misdemeanor arrests resulted in variable length jail stay. Felony arrests resulted in jail stay prior to trial followed by release, jail stay, or prison stay [31,32,35–37]. We did not explicitly model HIV, HCV, or treatment for substance use disorder during incarceration, but we did include healthcare costs specific to a jail or prison setting [31]. We assumed that transmission was sufficiently negligible not to model HIV and HCV transmission within jails or prisons; if anything, this biases against the diversion program. We assumed that treatment would not necessarily be resumed following release from incarceration [5,6]; instead, individuals re-entered the general, untreated population and were as likely to re-enroll in treatment as before incarceration. Fatal overdoses were more likely to occur in PWID during this transition, as drug tolerances become lower during incarceration [8–12]. Although there is substantial documentation of jails and prisons providing substandard care, there is also evidence that some individuals receive more continuous and effective healthcare while incarcerated [7]. We conservatively assumed no change in QALY value due to incarceration.

### Measure: Disease transmission, progression, awareness, and treatment

HIV or HCV transmission occurred between individuals who had a sexual or injecting partnership. We used a negative binomial distribution to characterize the number of partnerships an individual engages in [27,38]. Partnerships could be injection-based, sexual, or both. We assumed preferential mixing, with individuals in similar risk, age, and sexual orientation groups being more likely to form partnerships with each other.

Individual, disease, and transmission-route specific probabilities were calculated to determine the chance of infection for every at-risk individual at each time step [27,39,40]. Individuals became aware of their infection status through testing and, if infected, could receive HIV or HCV treatment. Adherence levels, reflecting daily uptake of medications, varied among individuals [27]. Adherence affected viral load and thus disease progression and transmission.

### Community programs

Community programs in the model included ART for HIV, HCV treatment, treatment for substance use disorder (SUDT), and needle and syringe programs (NSPs). PWID and PWUD were eligible to enroll in SUDT, which broadly included both drug rehabilitation and opioid agonist therapies. SUDT improved quality of life and reduced injection frequency for PWID, thus decreasing both disease transmission and overdose risk [8,41,42]. To enter an SUDT program, an individual had to join a waiting list. In keeping with observed patterns, we assumed that 35% of individuals on the waitlist dropped out every month [43,44]. PWID were eligible to use NSPs, which reduced disease transmission risk without reducing overdose risk [45]. Being enrolled in NSPs made it more likely for an individual to (attempt to) enroll in SUDT; likewise, being enrolled in SUDT made it more likely for an individual to be diagnosed as HIV-infected and to seek HIV or HCV treatment [27]. At any time, an individual could quit one, or both, programs. This was enforced by default when an individual committed a crime and moved to jail.

### Measure: Demographic transitions

Via SUDT, a small percentage of PWID and PWUD could transition to lower-risk groups (PWID to PWUD, or PWUD to general population) [42]. Individuals in the general population could spontaneously become PWUD, and PWUD could spontaneously become PWID [23,46,47]. Individuals entered the model at age 18, matured out of the model at age 75, and could die at any time step. Background mortality rates were age-, sex-, and race-specific [48]; additional risk came from drug use and disease status [49–51]. PWID and PWUD could overdose [9–12,41,52–54], and overdoses could be fatal or nonfatal [55,56].

### Intervention

Programs such as LEAD seek to connect individuals to housing, employment, and treatment opportunities [17,18]. Connection to housing and employment are likely the main pathway for the reduction in criminal activity associated with LEAD [20]. These were not explicitly modeled. Connection to community programs (NSP, SUDT, and HIV and HCV treatment) was explicitly tracked as part of our model, with downstream costs included in the healthcare ICER. Although enrollment in a diversion program likely improves quality of life, we conservatively assumed no change in QALY value associated with enrollment status. However, because we modeled the direct benefit of the diversion program in increasing engagement in community programs, we thereby captured indirect QALY benefits accruing from the diversion program. In the base case, we assumed that the diversion program doubled the probability of attempting to enroll in a community program and halved the probability of quitting such a program if enrolled. We explored a range of values in 2-way sensitivity analysis.

In intervention simulations, a PWID or PWUD committing a misdemeanor was eligible to enroll in the diversion program if not already enrolled; if he did enroll, he was not moved to jail [55,56]. Enrollment probability was assumed to be 25% in the base case and ranged between 10% and 75% in sensitivity analysis. At the time of next criminal activity (if it occurred), a diversion program-connected individual entered the criminal justice system but did not lose association with the diversion program unless the crime was a felony or the sentence duration exceeded 1 year [20,21]. Barring this, an individual remained associated with the diversion program for the remainder of the modeled time horizon. We attributed a 42% direct reduction in criminal activity to the diversion program, which is substantially lower than the 58% reduction observed for the LEAD program [20]. This is because additional, indirect benefits came from reduced criminal activity when individuals were enrolled in SUDT [57]. In our model, a 42% direct reduction in criminal activity approximated a 58% total reduction in criminal activity among those enrolled in the diversion program.

### Measure: Economic model

Program costs for an individual enrolled in the diversion program were estimated to be $899/month for the first 3 years and $532/month subsequently [20]. All other costs were taken from the literature or from King County–specific data. We distinguished costs that accrued from healthcare-related activities, including the healthcare-specific costs of incarceration, and those that accrued from all other aspects of the criminal justice system.

At the end of a model run, the costs (in 2016 US dollars) and QALYs associated with every individual at each time step were discounted to the present at 3% annually [58] and combined to calculate a single cost and QALY value over the modeled 10-year time horizon. Additionally, we projected remaining lifetime cost and QALY values for every individual alive in the model at the end of the run. These additional costs and QALYs, once discounted, were added to the final total for each trial run. Trial results were averaged to determine a final cost and QALY estimate for each scenario. We assessed intervention value by calculating incremental costs and QALYs with the diversion program compared to the status quo (with no diversion program) [59]. We calculated a healthcare ICER (healthcare system perspective) and an all-costs ICER (societal perspective) [59].

### Calibration

We manually calibrated the model to King County epidemiologic targets such as jail and prison populations; HCV and HIV incidence, prevalence, awareness, and treatment; and overdose deaths and HIV-related mortality, both in the population as a whole and in multiple population subgroups. A majority of input parameter values were calculated explicitly from published King County, Washington data or were well established in the literature on disease natural history and other infectious disease models. A subset of parameters had unknown or wide uncertainty intervals; we varied these in our calibration process.

For tractability, we took an incremental approach, manually varying subsets of model parameters to stabilize subsets of the model dynamics. We first calibrated parameters related to incarceration dynamics to replicate constant crime rates and jail and prison populations (Figs A-D in S2 Text). We then used a similar approach to calibrate community program enrollments (Figs E and F in S2 Text show results for the PWID population), which depended on incarceration rates but which we assumed could be isolated from disease dynamics. Disease incidence, awareness, and treatment all depended upon community program enrollments, so once we had adjusted those parameters appropriately, we calibrated to the remaining epidemiologic targets (Figs G-J in S2 Text show results for the PWID population). We began with HIV-related parameters, which involved interplay between risk groups, and having established those, adjusted HCV-related parameters, which were mostly internal to the PWID community. S2 Text provides full details of the calibration process.

## Results

### Main analysis

Table 3 summarizes health and demographic outcomes. In the base case (second row of table), the diversion program reduced cumulative HIV and HCV incidence by 3.4% (95% CI 2.7%–4.0%) and 3.3% (95% CI 3.1%–3.4%), respectively, over 10 years. Cumulative fatal overdoses decreased by 10.0% (95% CI 9.8%–10.8%) because of a combination of increased SUDT access and decreased jail cycling, and at the end of the 10-year time horizon, the daily jail population had reduced by 6.3% (95% CI 5.9%–6.7%). The public health benefit of the diversion program came both from a reduction in mortality (fewer overdoses and fewer deaths from HIV and HCV) and morbidity (fewer individuals infected with HIV or HCV and improved quality of life for individuals enrolled in the diversion program and community programs).

**Table 3:** Health and demographic outcomes: One-way sensitivity analysis on probability of enrollment in the diversion program.

	Percent reduction in					
Fraction of misdemeanor arrests that result in diversion	HIV incidence*	HCV incidence*	PWID fatal overdose*	Ending jail population*	ICER: Health System Perspective (Cost/QALY gained)^†^	ICER: Societal Perspective (Cost/QALY gained)^‡^
10%	1.4%	1.5%	4.9%	2.8%	$25,400	$3,700
	[0.8%, 2.0%]	[1.3%, 1.6%]	[4.4%, 5.4%]	[2.4%, 3.2%]	[$847, $178,000]	[Cost-saving, $115,000]
25%^**‡**^	3.4%	3.3%	10.0%	6.3%	$25,500	$6,200
	[2.7%, 4.0%]	[3.1%, 3.5%]	[9.8%, 10.8%]	[5.9%, 6.7%]	[$12,600, $48,600]	[Cost-saving, $24,300]
50%	5.1%	5.6%	15.1%	9.1%	$36,600	$13,800
	[4.5%, 5.7%]	[5.4%, 5.7%]	[14.6%, 15.6%]	[8.7%, 9.5%]	[$25,000, $54,000]	[$5,000, $27,000]
75%	7.1%	7.4%	18.7%	11.1%	$37,200	$14,700
	[6.4%, 7.7%]	[7.3%, 7.6%]	[18.2%, 19.2%]	[10.7%, 11.5%]	[$28,000, $50,000]	[$8,000, $24,000]

All results are relative to the case of no diversion program

*95% CIs were calculated for the difference in means between the status quo and intervention runs. Ranges presented here have been normalized by the mean of the status quo run.

†Ranges based on a best- and worst-case scenario as calculated from the extreme values for incremental costs and QALYs taken from their respective 95% CIs.

‡Base case analysis.

HCV, hepatitis C virus; HIV, human immunodeficiency virus; ICER = incremental cost-effectiveness ratio; QALY, quality-adjusted life year.

Table 4 presents economic results from the base case analysis. Over 10 years, 8,600 QALYs were gained. Incremental healthcare costs were $221 million, and incremental criminal justice savings (not including savings from healthcare delivered to the incarcerated) were $168 million, leading to an incremental societal cost of $53 million. When only healthcare costs were considered, the program cost $25,500 (95% CI $12,600–$48,600) per QALY gained (Fig K in S2 Text). When we took a societal perspective and included savings from the criminal justice system, the ICER reduced to $6,200 (95% CI cost-saving, $24,300) per QALY gained.

**Table 4:** Base case results: Costs and QALYs.

Scenario	Total Healthcare Costs (Billion)	Total Criminal Justice System Costs* (Billion)	Total Costs (Billion)	Total QALYs (Million)	Incremental Healthcare Costs (Million)^†^	Incremental Costs (Million)^†^	Incremental QALYs (Thousand)^†^	ICER: Health System Perspective (Cost/QALY Gained)^‡^	ICER: Societal Perspective (Cost/QALY Gained)^‡^
Status quo	$449.4	$29.0	$478.4	421.2	-	-	-	-	-
Diversion program	$449.6	$28.8	$478.5	421.3	$220.7	$53.2	8.6	$25,500	$6,200
					[$139.3, $302.0]	[Cost-saving, $149.1]	[6.2, 11.1]	[$12,600, $48,600]	[Cost-saving, $24,300]

*Excluding healthcare costs associated with incarceration, which are accounted for in the “Total Healthcare Costs” column.

^†^95% CIs were calculated for the difference in means between the status quo and intervention runs.

^‡^Ranges based on a best- and worst-case scenario as calculated from the extreme values for incremental costs and QALYs taken from their respective 95% CIs.

ICER = incremental cost-effectiveness ratio. QALY = quality-adjusted life year.

### Sensitivity analysis

We performed sensitivity analysis to examine the effects of the various assumptions and provide insight into the main drivers behind diversion program costs and benefits.

Table 3 assesses sensitivity of results to the fraction of eligible individuals with misdemeanor arrests who enrolled in the diversion program; this reflects both program capacity and participant willingness. We considered values of 10%, 25% (base case value), 50%, and 75%. As the enrollment probability increased, HIV and HCV transmission, fatal overdoses among PWID, and the size of the jailed population uniformly decreased. Despite slight decreases in projected value as enrollment increases, the program was cost-effective at all enrollment levels. This sensitivity analysis suggests that low or moderate enrollment levels can provide high value and that municipalities can continue to gain substantial health benefits and criminal justice system savings by expanding such a program to maximal enrollment.

Table 5 varies assumptions surrounding the indirectly modeled health benefits of the diversion program—namely, benefits that accrue from enrollment in community programs (NSP, SUDT, and HIV and HCV treatment). The base case assumed that an individual in the diversion program was, on average, twice as likely to attempt to enroll in community programs and half as likely to quit as an individual not enrolled in the diversion program. When combined, these two variables, connection to and retention in community programs, were a proxy for coverage of NSP, SUDT, and HIV and HCV treatment for individuals in the diversion program.

**Table 5:** Two-way sensitivity analysis on chance of joining and quitting community programs (NSP, SUDT, and HIV and HCV treatment): ICERs*.

		Multiplier for quitting community programs					
		1.00		0.50†		0.25	
		ICER: Healthcare System Perspective	ICER: Societal Perspective	ICER: Healthcare System Perspective	ICER: Societal Perspective	ICER: Healthcare System Perspective	ICER: Societal Perspective
**Multiplier for joining community programs**	**1.00**	$348,000	$340,000	$175,000	$162,000	$125,000	$108,000
	**1.50**	$108,000	$95,000	$106,000	$77,000	$40,000	$22,000
	**2.00†**	$65,000	$44,000	$25,000	$6,000	$11,000	Cost-saving
	**2.50**	$23,000	$6,000	$8,000	Cost-saving	Cost-saving	Cost-saving
	**3.00**	$11,000	Cost-saving	Cost-saving	Cost-saving	Cost-saving	Cost-saving

*All results (and multipliers) are relative to the case of no diversion program. ICERs rounded to the nearest $1,000.

†Base case value.

HCV, hepatitis C virus; HIV, human immunodeficiency virus; ICER, incremental cost-effectiveness ratio; NSP, needle and syringe programs; SUDT, treatment for substance use disorder.

We considered multipliers ranging from no change (1.0) to high increase in the chance of enrolling (3.0) and high decrease in the chance of quitting (0.25). As expected, when we kept one variable fixed at no change (1.0) and increased the other, the value of the diversion program increased monotonically. However, Table 5 shows that doubling the probability of enrolling in a community program provided far more value than halving the chance of quitting such a program. This because individuals must first enroll in a program before a reduction in the chance of quitting can provide substantial benefit.

When the diversion program did not increase an individual’s propensity to attempt to enroll in community programs (multiplier 1.0, first row of Table 5), the ICER was significantly higher than in the base case, indicating that the value of the diversion program is closely tied to increased engagement in community programs. When highly effective at increasing engagement in community programs, a diversion program can even be cost-saving.

Our base case analysis assumed that the diversion program would cost $899 per year for the first 3 years and $532 per year in subsequent years for each participant, based on the cost of Seattle’s LEAD program [20]. Other diversion programs may have different costs. If the cost of the program is 50% higher than we assumed, then the ICER from the health system perspective increases to $88,800, and the ICER from the societal perspective increases to $67,700. If the costs are twice as high as in the base case, these values increase to $140,800 and $116,200, respectively, indicating that such a program would likely still be considered cost-effective.

Our model is calibrated to King County, Washington. For the base case, we estimated that 80% of PWID partners would be PWID, 18% would be PWUD, and 2% would be from other population groups and that 60% of PWUD partners would be PWUD [27,60], and we calibrated our model accordingly. If PWID and PWUD have a greater propensity to mix with the general population (with these fractions changing to 70%, 20%, 10%, and 50%, respectively), then the ICER increases slightly to $29,100 for the health system perspective and $12,700 for the societal perspective. Similarly, HIV and HCV prevalence and incidence may be different in other settings, affecting some of the potential health benefits of a diversion program. If HIV and HCV transmission are 25% lower than in the base case, then the ICER increases to $49,900 from the health system perspective and to $17,900 from the societal perspective. Finally, we varied the weekly chance of an identified crime, increasing this rate by 20% and decreasing it by 20%, and found that such changes had only a modest effect on cost-effectiveness, with the ICER from the healthcare perspective remaining below $47,000 and the ICER from the societal perspective below $28,000.

## Discussion

Our analysis showed that a diversion program for low-level drug offenders, as modeled based on the diversion program and setting in Seattle, is likely to substantially reduce spending in the criminal justice system while moderately increasing spending in the healthcare sector. When considering healthcare costs only, we found that the diversion program was highly cost-effective at $25,500 ($12,600, $48,600) per QALY gained. Including savings from reduced incarceration improved the ICER to $6,200 (cost-saving, $24,300) per QALY gained. Additionally, the diversion program reduced the jailed population size and provided significant health benefits, decreasing fatal overdoses among PWID and reducing HIV and HCV incidence. Critically, we found that the effectiveness of a diversion program in connecting participants to community programs such as SUDTs, NSPs, and treatment for HIV and HCV greatly affects cost-effectiveness.

As a relatively new initiative, fewer data are available for diversion programs than for other, more established interventions aimed at people with substance use disorder. When we encountered uncertainty for program parameters in our model, we tended toward conservative estimates that would bias us against such a program. For example, we did not include any direct QALY increase associated with diversion program enrollment, despite the fact that such a program would help clients obtain housing and employment, among other benefits [18]. We did not include any direct QALY decrease associated with incarceration, nor did we model HIV and HCV transmission among incarcerated individuals. In reality, we would expect the decrease in jail population due to the diversion program to further decrease infectious disease morbidity. The LEAD program decreased both overall recidivism and the number of felony arrests [20]. We modeled an overall reduction in crime but did not include changes in type of crime. Because felony arrests are more expensive downstream, our analysis likely understates the cost savings of such a program. Finally, our analysis used data from King County, which has steady infection rates, already high ART coverage, and active NSP services. Had we modeled a diversion program in an area with a growing HIV or HCV epidemic, for instance, we might have seen further benefit from the diversion program-to-community interventions pathway. Nonetheless, even with conservative assumptions, we found the diversion program to be cost-effective both as a healthcare intervention and more broadly as a societal intervention.

Like any model of a complex system, our analysis has limitations. While attempting to capture key aspects of the diversion program, inevitably, we had to simplify pathways in the healthcare and criminal justice systems for tractability. For instance, we did not model the “social contacts” pathway through which individuals can be referred to a diversion program without first being diverted from a crime [17]. As another example, because of the lack of data, we assumed that average behavior of individuals enrolled in the diversion program does not change as a function of the enrollment level. As diversion programs evolve, more nuanced analyses can be performed. We modeled SUDT at an aggregate level with an average reduction in frequency of injection and rate of overdose deaths, an average increase in quality of life, and a certain fraction of individuals ceasing drug use. Depending on the form of SUDT that is offered, other parameter values could be used to reflect SUDT efficacy. Additionally, our analysis from the societal perspective includes costs of the diversion program, healthcare costs, and criminal justice system costs but does not include other potentially relevant but less quantifiable costs such as costs associated with homelessness, unemployment, and breakdown in family structure.

Parameter uncertainty is always a challenge for public health models. One advantage of modeling a county was that data taken from multiple sources were measuring the same population, enabling us to infer key demographic parameters and check consistency across subgroups. Although there is perhaps more uncertainty and variability in disease natural history parameters, our HIV and HCV models closely reflect published studies [27,39,49,51,61]. We also introduced a high level of detail into the model, allowing for heterogeneity, so that no single aggregate parameter unduly determined disease transmission or progression. Monte Carlo methods allowed for uncorrelated variability in model parameters between runs as a probabilistic sensitivity analysis in a dynamic compartmental model would, and running 2,500 trials for each scenario reduced noise. Nonetheless, our findings are subject to parameter uncertainty and other limitations discussed here.

Another challenge in such an analysis is structural uncertainty; that is, understanding how model results depend on the type of model that is used [62]. We used a microsimulation model for this analysis, as it allowed us to capture the spread of 2 infectious diseases in multiple risk groups and with multiple health and incarceration states, analysis that would be difficult to perform using a compartmental model. In other work, we performed a structural sensitivity analysis in which we created 7 simpler versions of our model, all calibrated to King County, Washington epidemiologic targets, which we used to evaluate a hypothetical HIV vaccine [63]. The models varied over 2 dimensions: parameter complexity (e.g., the inclusion of age and HCV comorbidity) and contact/simulation complexity (e.g., compartmental versus network models). The analysis showed that simple compartmental models tended to overestimate the effect of the intervention and that contact/simulation complexity had a greater effect than parameter complexity on estimates of intervention effectiveness. This suggests that our microsimulation model may provide reasonably accurate estimates of intervention impact.

A 2016 UNAIDS report found that the status quo of criminalizing drug abuse is failing from the public safety and public health perspectives [1]. Our study demonstrates that these perspectives can be aligned. Our analysis includes health and system dynamics that are critical to PWID and PWUD outcomes but are rarely included in one model [64] and is uniquely positioned to assess the mechanisms by which a diversion program accrues both costs and benefits. We found that diversion programs for drug offenders can provide high value and have the potential not only to reduce incarceration and its associated costs but also to avert overdose deaths and improve quality of life for PWID and PWUD, as well as the broader population (through reduced HIV and HCV transmission). The spread of infectious disease and the global overcrowding of jails and prisons suggest that, more than ever, effective, cost-effective, and innovative approaches are required to address these crises. In addition to evaluating one such intervention, our study illustrates how interdisciplinary modeling can provide valuable insight into complex public health burdens.

## Supporting information

S1 Text CHEERS checklist. CHEERS, Consolidated Health Economic Evaluation Reporting System.
(DOCX)

S2 Text Model details, input parameters, model calibration results, and cost-effectiveness plane. (DOCX)

## Abbreviations

CHEERS: Consolidated Health Economic Evaluation Reporting System
HCV: hepatitis C virus
ICER: incremental cost-effectiveness ratio
LEAD: Law Enforcement Assisted Diversion
NSP: needle and syringe programs
PWID: people who inject drugs
PWUD: people who use drugs but do not inject
QALY: quality-adjusted life year
SUDT: treatment for substance use disorder
UNAIDS: United Nations AIDS Programme
