# Supplementary material for: Health outcomes and cost-effectiveness of diversion programs for low-level drug offenders: A model-based analysis
Source: PLoS Med. 2020 Oct 13;17(10):e1003239. doi: 10.1371/journal.pmed.1003239 (PMC7553283; doi:10.1371/journal.pmed.1003239)
Supplement: S2 Text — (DOCX) [file pmed.1003239.s002.docx]

**Supplement S2 – Health Outcomes and Cost-Effectiveness of Diversion Programs for Low-Level Drug Offenders: A Model-Based Analysis**

Cora L. Bernard, Isabelle J. Rao, Konner K. Robison, Margaret L. Brandeau

**Overview …………………………………………………………………………………. 2**

**Model Population ………………………………………………………………………… 2**

***Demographics* …………………………………………………………………………. 2**

***HIV, Initial Conditions* ……………………………………………………………..… 4**

***HCV, Initial Conditions* ………………………………………………………….…… 6**

***Partnerships* ………………………………………………………….………..……… 8**

***Incarceration* ………………………………………………………………….…...…. 10**

***Community Programs* ……………………………………….………….………….… 11**

**Model Simulation ……………………………………………………………………….. 12**

***Criminal Activity and Incarceration* …………………………………………………. 12**

***Simulation of Partnerships* …………………………………………………….….… 14**

***Disease Transmission* …………………………………………………………...…… 15**

***Disease Progression* …………………………………………………………….…… 18**

***Disease Awareness and Treatment* …………………………………………….……. 20**

***Community Programs* ……………………………………………………………..… 23**

***Demographic Transitions* ………………………………………………….………… 24**

***Mortality ………………*……………………………………………………………… 25**

**Model Calibration ……………………………………………………..……………….. 25**

**Economic Model ……………………………………………………………………….. 28**

# Overview

The model is a network model with discrete-time stochastic simulation. Parameterization and programming of the base case model can be divided into two parts: 1) establishing the initial model population; 2) implementing and recording the transitions of that changing population at each time step. The first part, covered in the section Model Population below, involves identifying the relevant characteristics to include for each individual, the “creation” of the individuals in the model, and the positioning of each individual within the appropriate initial structure (un-incarcerated, jail, or prison). The second part is covered in the Model Simulation section. The Model Calibration section details how the model was calibrated to epidemiologic targets and provides outputs from simulations of the status quo. The Economic Model section provides information on cost data.

The model was programmed in Python™ version 2.7.10. Simulations were run on the Barley and Sherlock clusters at Stanford University, which are maintained and made available by the Stanford Research Computing Center. Some parts of this appendix appear as a technical supplement in [1].

# Model Population

## *Demographics*

We performed an extensive literature review to gather background information and parameter values regarding population demographics and risk behaviors in King County, Washington. Based on substantial differences in incarceration rates, disease prevalence and natural history, partner selection, and background mortality rates, we identified key individual characteristics to include in the model: risk group (person who injects drugs (PWID), person who abuses but does not inject illicit drugs (PWUD), and non-drug user (low-risk, LR)); race (black, white, or other); sex (male or female); sexual orientation (men who have sex with men (MSM), heterosexual male, heterosexual female); and age group (18-19, 20-29, 30-39, 40-49, 50-59, or 60-74 years old).

We assigned characteristics sequentially to account for dependencies. For instance, an individual *i* was first assigned a risk group by drawing a random number $v_{i}$ $\in\left[ 0,1 \right]$. Consider the parameters $\alpha_{PWID}=p_{PWID}, \alpha_{PWUD}=p_{PWID}+p_{PWUD}$ where $p_{j}$ is the proportion of the population in group *j*. (For low-risk individuals, we have ${p_{LR}=1-p}_{PWID}-p_{PWUD}$). When ${v \leq\alpha}_{PWID}$, we assigned risk group PWID to individual *i*. When ${\alpha_{PWID} <v \leq\alpha}_{PWUD}$, we assigned risk group PWUD to individual *i*. Otherwise, the individual *i* was assigned to the low-risk group.

Individual *i* was then assigned race in a similar manner, where now the distribution of race depended on the risk group, *j*, and the relevant comparison parameters were $\alpha_{black|j}=p_{black|j}, \alpha_{white|j}=p_{black|j}+p_{white|j}$, where $p_{other|j}= {1-p}_{black|j}+p_{white|j}$. In the absence of data to the contrary, race and sex were assumed to be independent so that sex was assigned only on the basis of risk group. Other characteristics were assigned using the same process of drawing a random number and comparing it to the calculated parameters reflecting the outcomes of previous draws.

Given the nature of the source data, in practice we used an assortment of conditional probabilities to distribute characteristics appropriately, so that the order of assignment, while reflecting the formats in which data were collected and presented, was not necessarily intuitive. For instance, the marker for HIV infection was assigned based on risk group, race, and sex. Sexual orientation was then assigned based on risk group, race, sex, and HIV status. Age was assigned according to risk group, HIV status, and sexual orientation. Finally, hepatitis C virus (HCV) status was assigned according to risk group, sex, and age. Because of the high rates of HCV among PWID [2-4], we independently assigned HIV and HCV infection to each individual, using risk group as the primary correlate of each which, after assignment, gave a rate of HIV/HCV co-infection comparable to King County estimates [5].

Table A provides demographic parameter values and data sources. Further information on HIV (*HIV, Initial Conditions*) and HCV (*HCV, Initial Conditions*) assignment can be found in the respective subsections below.

King County, Washington has an approximate population of 1,390,300. For computational tractability, we simulated our model on a population roughly 10% of the total population while keeping demographic distributions consistent with King County as a whole. After instantiating each of the 140,000 individuals in the model, we assigned sexual and injecting partnerships; this algorithm is described in greater detail in *Partnerships*. We then assigned initial incarceration status according to the risk group, race, sex, and age group of each individual. Further detail is provided in *Incarceration*. Finally, unincarcerated individuals could be connected to programs such as needle/syringe exchange programs (NSP) or treatment for substance use disorder (SUDT); this is discussed below in *Community Programs.*

## *HIV, Initial Conditions*

Estimates of HIV prevalence in each risk group, race, and sex category were taken from King County, Washington-specific reports [2]. After drawing a random number to assign infection status, we determined the awareness status, treatment status, and CD4 count and viral load based on distributions observed in the literature. To identify the appropriate parameters to use for an individual, we used a hierarchical stratification of risk factors. If an individual was PWID, then all parameters associated with PWID (e.g., probability of being on treatment) were applied to that person, regardless of other risk characteristics (e.g., sexual orientation). If the individual was MSM, but not PWID, that became his primary risk factor. Otherwise, individuals were assigned parameters in keeping with heterosexual-specific data from King County, Washington.

Every individual, regardless of whether infected with HIV at time 0, was assigned a set of baseline characteristics, which were activated if/when infection occurred. Some characteristics inherent to an individual did not change throughout the simulation. These included an adherence parameter, which determined an individual’s likelihood of adhering to HIV antiretroviral therapy (ART) or HCV treatment, a viral load set point, and a CD4 count. Only those individuals infected at time 0 were assigned awareness and treatment status.

In keeping with a published microsimulation of HIV transmission [6], adherence was assigned on a 1-5 integer scale, where an adherence level of 5 corresponded to $\geq90\%$ adherence, decreasing to 70–89% at level 4, 50–69% at level 3, 30–49% at level 2, and 0–29% at level 1. We assumed that 60% of individuals had level 5 adherence, and 10% of individuals were assigned to each remaining level [6].

We assigned awareness of HIV status based solely on risk group [2, 7] and additionally assumed that individuals in the acute phase of HIV at time 0 (a small proportion of the total infected population [6]) could not yet be diagnosed. For those who were aware of their HIV infection, we determined, based on King County, Washington records, the probability of having AIDS by risk group and sex/sexual orientation [2]. If AIDS was assigned by a random draw, the individual was given a CD4 count uniformly selected to be between 5 and 200 cells/mm^3^. Otherwise, the CD4 count was uniformly selected to be between 201 and 700 cells/mm^3^.

For individuals aware of their HIV infection, we next assigned ART treatment status by PWID, MSM, or low-risk characteristic [2]. We assumed that no individual currently incarcerated could be on treatment. Individuals assigned to be on ART who had an adherence parameter of level 4 or 5 were considered to be virally suppressed. An individual’s viral load set point was defined as the viral load they would have in the absence of treatment; we drew this value from a truncated normal distribution [8]. For individuals who were virally suppressed, a current viral load was drawn from another truncated normal distribution, which was capped at 2.3 log_10_ copies/ml [2].

For individuals who were not aware of their HIV infection, and could not therefore be on ART, we assumed their current viral load was the same as their viral set point. Their CD4 count was assigned based on a distribution for unaware, HIV-infected individuals found in the literature [8]. Individuals in the acute HIV stage had a CD4 count uniformly assigned to be between 500 and 1600 cells/mm^3^ [8].

Table B provides parameter values and data sources related to the assignment of initial conditions for HIV-infected individuals.

## *HCV, Initial Conditions*

We included 9 possible states related to HCV: uninfected, recovered, acute infection, no fibrosis (F0), portal fibrosis (F1), periportal fibrosis (F2), bridging fibrosis (F3), compensated cirrhosis (F4), and decompensated cirrhosis (ESLD, or end-stage liver disease) [9]. To initialize the population we used data on HCV prevalence in each age, risk, and sex group [3-5, 10] to assign each individual a probability of HCV infection at time 0. Broad HCV prevalence estimates for lower-risk groups were available [3, 10], but for PWID, data were often presented in terms of the presence of HCV antibodies in the blood, and did not differentiate between current and prior infection [3-5]. Using an estimate of the number of individuals who spontaneously clear the infection [9], the fraction of PWID who have received care [11, 12], and the fraction of those who have successfully completed care [4], we calculated a percentage for each age group that we estimated to be “cured” and used it to determine the proportion of individuals who were still actively infected.

Once we determined whether an individual was HCV-infected, we assigned 1% of HCV-infected individuals to the acute infection stage, which means they had been infected with HCV for less than 6 months. (The 1% probability was chosen during calibration to be consistent with rates of progression to chronic infection.) All other individuals were considered chronically infected. Because we did not have epidemiologic estimates for the distribution of chronic HCV stages at the desired level of detail, we used progression probabilities taken from the literature [13] and a recursive algorithm to calculate the distribution of individuals among the HCV disease stages (F0, F1, F2, F3, and ESLD) given years since HCV infection, sex, age, years since HIV infection, and HIV treatment status. We checked the validity of this algorithm against populations in which distribution data was available [9].

We then used our calculated HCV disease stage distributions as input data. For each individual we uniformly drew a random integer between 0 and (individual’s age – 18) and multiplied it by 12 to approximate months since infection and used this along with other already assigned demographic characteristics to assign each individual to a plausible HCV disease stage.

Each HCV infection was associated with a genotype, either G2/G3 or G1/G4, with probability consistent with the genotype distribution in the general population [14]. We assumed that individuals with a G2/G3 genotype infection would require 6 months of treatment to clear the infection, while individuals with a G1/G4 genotype infection would require 12 months of treatment [9]. Due to high quit rates in HCV treatment, we randomly assigned a small proportion of infected individuals to be on treatment at time 0 [9]. For those on treatment, we uniformly selected an integer between 0 and the required months on treatment to mark how far into their course of treatment they already were. We assumed that no individual currently incarcerated could be on treatment.

Similar to the inherent characteristics discussed in *HIV, Initial Conditions,* we assigned certain unchangeable properties to each individual, regardless of current HCV infection status, which would become relevant if/when an HCV infection occurred. We assigned a monthly probability of quitting HCV treatment, which correlated to an individual’s ART adherence parameter (e.g., a higher adherence level meant a lower chance of quitting). We also randomly assigned individuals as being CC-type or non-CC-type (these are variants of the human IL28B gene), with the probability of assignment based on race [15]. CC-type individuals are more likely to have a sustained virologic response following HCV treatment (i.e., they are more likely to be “cured” if they complete treatment) [9].

Table C provides parameter values and data sources related to the assignment of initial conditions for individuals infected with HCV.

## *Partnerships*

We used a negative binomial distribution to characterize the number of partnerships per individual [6], which has previously been found to best characterize social networks for infectious disease spread [16]. According to risk group, age, sex, and sexual orientation, each individual *i* was assigned the parameters *p_i_* $\in$ (0,1), the probability of success per trial, and *r_i-_* $\in$ $\mathbb{N}$*,* the number of failures permitted before the trials are stopped. For tractability, we track a sub-network of partnerships. Degree 0 individuals are infected with HIV and/or HCV and can transmit one or both diseases to their uninfected partners. (An exception is the group uninfected with HIV but infected with acute HCV. We assumed that they cannot transmit HCV currently but we assigned them as Degree 0 to anticipate their shortly becoming infectious.) Degree 1 individuals are uninfected with both HIV and HCV and cannot transmit a disease but are linked by a partnership to a Degree 1 individual. Degree 2 individuals are similarly uninfected and are linked to a Degree 1 individual while having no Degree 0 partners. In this way we assigned partnerships to anticipate the spread of disease without having to assign all possible partners to all uninfected individuals (the majority of the model’s population).

We used the following algorithm to assign partnerships at time 0. Let *S* be the set of all individuals awaiting the assignment of partners. Let *A* be the set of all available partners and let *U* be the set of all individuals in the modeled population. Let *D_d_* be the set of all individuals who are of degree *d* in the network. (In particular, *D_0_* is the set of all individuals infected with HIV and/or HCV). Let $k_{i}^{0}$ be the number of partners assigned to individual *i.*

1. Set *S* = *D_0_*. Set *A=U*. Set $k_{i}^{0}=0 \forall i$.
2. While *S* is non-empty, select $i \in S. S -= i.$ $A -= i.$ (That is, remove *i* from the pool of individuals needing partner assignments as well as from the pool of individuals available to be assigned partners.) Assign partners to *i*:
   1. Simulate the flip of a weighted, random coin that is heads with probability *p_i_*, until *r_i_* tails have occurred. The number of resulting heads, $h_{i}^{0}$, is the number of partners individual *i* should be assigned in the absence of incarceration at time 0. Note that $h_{i}^{0}\sim NB\left( p_{i},r_{i} \right)$and therefore for $n\in$ $\mathbb{N}$, $P\left( h_{i}^{0}=n \right)= \frac{\left( n+r_{i}-1 \right)!}{\left( r_{i}-1 \right)!n!}{p_{i}}^{r_{i}}{(1-p_{i})}^{n}$.
   2. For *n=* 1,*…,*${max(0,h}_{i}^{0}-k_{i}^{0})$*:*
      1. To determine the appropriate demographics of *i*’s *n*^th^ partner, draw a series of random numbers to determine whether the partner is heterosexual male, heterosexual female, or MSM; whether the partner is PWID, PWID, or low-risk; and which age group the partner is in [6, 17, 18]. The likelihoods of these assignments are based on individual *i*’s characteristics.
      2. Randomly select a partner *j* from $A \cap A_{j}$, where $A_{j}$ is the set of individuals in the model with the selected sex/sexual orientation, risk group, and age characteristics. Assign a link between *i* and *j*. Partnerships may be casual or main [19]. If both *i* and *j* are PWID, the link may be a sexual partnership, an injecting partnership, or both [6].
      3. $k_{i}^{0} += 1,k_{j}^{0} += 1$.
      4. If $i \in D_{0}, j \notin D_{0}$, then $D_{1}+=$ *j* and $S +=j$. If $i \in D_{1}, j\notin D_{0}, j\notin D_{1}$, then $D_{2}+=$ *j* and $S +=j$.

Table D provides parameter values and data sources related to the assignment of partnerships in the model.

## *Incarceration*

At the start of the model run, and at any time thereafter, an individual can be in one of three locations: jail, prison, or unincarcerated. We assumed that all individuals in the prison population are serving a felony sentence. According to risk group, sex, and race, we sampled an initial prison population from the King County population [20-24] and assigned sentence length, *s*, in months to each individual [21, 24], then drew a random integer from $r \in[1,s]$ to establish the remaining number of months of their sentence to be served. We assumed the prison system was state run and would include individuals from multiple counties beyond King County; we only tracked the population associated with King County.

Individuals in the jail population might be awaiting a court proceeding regarding a misdemeanor crime, serving a misdemeanor sentence, awaiting a court proceeding regarding a felony crime, or serving a felony sentence. According to risk group, sex, and race, we sampled an initial jail population from the King County population [20-22, 24] and then assigned the type of crime and pre/post-sentencing status to each individual according to distributions specific to Washington State and, where possible, King County specifically [21, 24, 25]. After determining crime and sentencing status, we assigned *r*, the number of months remaining in the sentence or until the sentencing is determined as we did for the prison population.

To be conservative about the public health effects of the incarceration system, we assumed that no HIV or HCV transmission occurs in jail or prison. We assumed that individuals in prison have no sexual or injecting partners and we disconnect from their assigned partners at the time of sentencing or at time 0. Because the majority of jail stays for misdemeanor crimes are less than one week in duration [21], we do not automatically disconnect jailed individuals from their partners. Rather, we assumed no partner contact for all individuals while in jail but only officially disconnected partnerships at the time of sentencing or at time 0 if an individual’s jail stay will exceed 1 year.

Table E provides parameter values and data sources related to the initial conditions of incarceration in the model.

## *Community Programs*

Community programs in the model include ART for HIV, HCV treatment, treatment for substance use disorder (SUDT), and needle and syringe programs (NSP) for PWID. PWID in the model are eligible to use NSP, which reduce the frequency of sharing injection equipment [26], and SUDT, which we assumed reduces the frequency of injections [27]. SUDT broadly includes opioid agonist therapies, such as methadone maintenance and buprenorphine maintenance therapies, as well as drug rehabilitation programs. PWUD can also be enrolled in SUDT. Where applicable, we assumed that PWID using NSP are more likely to enroll in and less likely to quit SUDT than PWID who do not use NSP, and individuals receiving SUDT are more likely to initiate and less likely to stop ART than individuals not receiving SUDT [6]. We assigned “membership” to each of these two programs by back calculating distributions (e.g., given that an individual is on ART, how likely is it that the person is receiving SUDT) so that, after assignment, initial enrollment was consistent with these assumptions.

In all runs, both base case and diversion program scenarios, the model began with no enrollment in the diversion program. Table F provides parameter values and data sources related to the initial enrollment of PWID and PWUD in community programs.

# Model Simulation

The model runs on weekly time steps to accommodate the frequency of criminal activity, the duration of sentences, and jail population turnover. All other transitions occur once every four weeks, i.e., on a monthly basis. Transitions occur in a fixed cascade so that transitions higher up in the cascade may prohibit or change the likelihood of further transitions during that time step. For instance, mortality happens at the beginning of the week and individuals who die are then ineligible for other transitions. Disease infection occurs before disease progression, and individuals who are newly infected have zero probability of progressing in their disease during the first month of their infection. Individuals who commit identified crimes may have their disease progress but may not infect their partners that week, etc. The transitions are presented in this section in an order chosen for clarity to the reader and are not necessarily in the order they occur in the model.

## *Criminal Activity and Incarceration*

We assumed a fixed, low-risk incarcerated population; that is, the initial low-risk population in jail and prison remain in these locations throughout the model run. They do not, therefore, represent actual modeled individuals but instead guarantee consistent demographics, while PWID and PWUD cycle in and out of jail and prison. Crimes by these populations are committed and identified in the model with a probability that is dependent on individual age, race, sex, and risk group. We assumed no difference in crime type and sentencing length by these identifiers so that the numbers of crimes committed annually by each subpopulation is proportional to the current incarcerated population breakdown [20-22, 24]. To calculate the weekly probability of identified criminal activity, we took the total annual number of crimes [21], the percent committed by PWID and PWUD, which we assumed to be roughly equivalent to the percent of the incarcerated population who are PWID and PWUD [20], the percent of these crimes committed by a specific age, race, sex group [20-22, 24], and divided by the total number population size of this age, sex, race, and risk group. Because SUDT reduces the need to get high through illegal sources, we associated SUDT with lowered criminal activity [28]. For individuals not on SUDT, we raised the probability of criminal activity so that net criminal activity across each age, sex, race, and risk group agreed with the original calculation.

If a crime is committed, it is assigned to be either a felony or a misdemeanor, consistent with the relative frequency of such events in Washington State [21]. A PWID or PWUD committing a misdemeanor crime is eligible to enroll in the diversion program if they are not already enrolled and, if they do enroll, they are not moved to jail (see *Diversion Program*). A very small percentage of misdemeanor crimes are assigned to drug court, from which we assume an even smaller percent of PWID and PWUD “graduate” to low-risk groups [29]. Otherwise, all individuals who are identified after committing a crime, regardless of the severity of their alleged crime (misdemeanor or felony), will be booked into jail [21]. At this time, an individual quits all treatments he or she is receiving and loses enrollment in community programs.

If the crime is a misdemeanor, a sentence time, which includes possible court proceedings, is assigned according to a Washington State sentencing distribution [21]. If the crime is a felony, the individual awaits trial in jail for a number of weeks according to a pre-sentencing distribution [30]. After a trial, some percentage of accused felons return to the unincarcerated population while others are given a sentence, which will be served either in jail or prison. Those with sentence lengths of a year or more are always sent to prison [21]. The outcome of the trial and the location and duration of the sentence are all assigned probabilities consistent with data from Washington State’s incarceration system [21]. Under our assumptions, individuals heading to prison disconnect from their sexual and injecting partners at the time of sentencing. We assumed that individuals who are not disconnected from their partners would not have sexual or injection-based contact with them while incarcerated.

Each week, every individual’s remaining sentence length is updated ($r-=1$). Individuals reaching the end of their sentence are released. Given the difficulty of transitioning from jail or prison back to normal life, we assumed that there is no automatic reconnection to disease treatment or community programs [31]. At the time of release, PWID are more likely to overdose [32-36], as we discuss further in *Mortality*. Table G provides parameter values and data sources related to incarceration transitions.

## *Simulation of Partnerships*

At every subsequent time step at which partnerships are modified we followed a similar algorithm to that outlined for *t=0* with slight modifications to steps b) ii and iii. In keeping with Marshall et al. [6] we simulated the dissolution and formation of new partnerships in a consistent, stylized way by allowing individuals to drop partners and gain new ones. As before, for individual $i \in S$, the number of partners to be assigned at time *t* is $h_{i}^{t}\sim NB\left( p_{i},r_{i} \right).$ Let$P_{i}$ be the set of partners that individual *i* has from time step *­*$t-1$, where ${len\left( P_{i} \right)= k}_{i}^{t-1}$, the number of assigned partners from time $t-1$*.* We begin with ${k_{i}^{t}= k}_{i}^{t-1} \forall i.$

- If $h_{i}^{t}$ < $k_{i}^{t}$, randomly select ${j=1,\ldots,min \left( len(P_{i}\cap A \right), k}_{i}^{t}-h_{i}^{t})$ partners where $j\in P_{i} \cap A$, to disconnect from *i.* For each *j* selected, $k_{i}^{t}-=1$ and $k_{j}^{t}-=1$.
- If $h_{i}^{t}$ > $k_{i}^{t}$, randomly select ${j=1,\ldots,min \left( len(A- P_{i} \right), h}_{i}^{t}-k_{i}^{t})$ partners where $j\in{A-P}_{i}$, to connect for *i.* For each *j* selected, $k_{i}^{t}+=1$ and $k_{j}^{t}+=1$.

Table D provides parameter values and data sources related to the assignment of partnerships in the model.

## *Disease Transmission*

HIV or HCV can be transmitted if two conditions are met. First, injection- or sexual-based contact must be made. (This is predicated upon a partnership existing between the uninfected person and an infected person and both partners being unincarcerated. We assumed, conservatively, that no transmission occurs within the incarceration system.) Second, given that contact is made, infection transfer must happen. The contact at time *t* occurs at a frequency, ${N(t)}_{ij}$, that depends upon characteristics of both the uninfected and the infected partner, *i* and *j*, respectively, and infection transfer occurs with a probability, $p_{ij}$, that depends on these characteristics as well as properties of the relevant disease. We model infection transfer as a Bernoulli process [37]. The probability of infection in a given month = 1 – probability no infection transfer in one month, where the latter probability is $\binom{{N(t)}_{ij}}{0}={(1-p_{ij})}^{{N(t)}_{ij}}$. The calculations for infection are adapted from previous models [27, 38].

The frequency of sexual contact is $N^{S}$, the average number of sexual encounters between two partners in one month [39]. The frequency of injection-based contact, ${N(t)}_{ij}^{I}$, depends on the number of injections between two partners in one month, which varies over time. To calculate this quantity, we begin with $N^{I}$, the average number of injections that a PWID makes per month [14]. The number of injections made by individual *i* is $N_{i}^{I}=\frac{{{\alpha_{share}\alpha}_{NSP}\alpha_{SAT}N}^{I}}{k_{i}^{t}}$, where the $\alpha$ parameters are multipliers less than 1 that specify that in the absence of NSP not all injections are shared ($\alpha_{share})$ or that reduce the frequency of sharing injecting equipment from NSP ($\alpha_{NSP}$) [26] or the frequency of injecting from SUDT ($\alpha_{SAT}$) [27]. We divide by $k_{i}^{t}$, the number of partners connected to *i*, because we assume that individuals with *n* injecting partners will not inject *n* more times per month than individuals with only 1 injecting partner. Finally, we calculate ${N(t)}_{ij}^{I}=\frac{N_{i}^{I}+N_{j}^{I}}{2}$.

We calculate the probability of infection transfer as follows: $p_{ij}= p_{j}(1-u_{ij}\kappa)$, where *p* is the disease-specific transmission probability given the stage of disease and/or treatment status of infected partner *j*, $u_{ij}$ is the probability that a condom is used in the partnership, and $\kappa$ is the effectiveness of the condom. The quantity $u_{ij}$ is the average of $u_{i}$and $u_{j}$, which are each determined by the individual’s risk group, sex, and sexual orientation and, additionally, whether they have HIV and are aware of it, in which case we assumed a modification of behavior [6, 7, 19, 40]. The value of the parameter $\kappa$ is disease-specific [14, 27].

For HIV, $p_{j}$ depends on viral load, the type of contact, either sexual or injection-based and, if sexual, the sex and sexual orientation of the partners (i.e., MSM to MSM, heterosexual female to heterosexual male, and heterosexual male to heterosexual female) [6, 8, 41]. Monteiro et al. [6] found that adding in viral load heterogeneity, rather than basing infection probabilities on CD4 count, reduced bias in predictive models of HIV transmission. To reduce calculation time, we did not calculate the probability directly from the individual viral load; rather, we placed individuals in viral load range blocks and used pre-calculated values based on those. The one exception to this is when the HIV infection is acute or the CD4 count has dropped below the AIDS threshold and the individual is not on effective treatment (i.e., treatment with a sufficiently high adherence level), in which case we used separate pre-calculated values for these highly-infectious stages [6, 8, 41].

The infection transfer probability for HIV does not directly depend on whether the infected partner is on ART or not. However, since viral load and HIV stage evolve as functions of treatment (see *HIV Progression*), the infection transfer probability for HIV is indirectly affected by treatment. For HCV, the infection transfer probability directly depends on whether the individual is receiving HCV treatment. This latter probability does not depend on the HCV disease stage, as long as the HCV infection is chronic, nor are sexual transmission parameters dependent on sex and sexual orientation [14, 42, 43]. The probability of HCV infection transfer is therefore a function only of the type of contact and whether the infected individual is receiving HCV treatment [14]. We made the assumption that the number of strains of HCV meant that previous antibodies from prior infection and recovery would not protect individuals from re-infection, so we did not take into account prior resolved infection when determining HCV infection transfer probability.

An uninfected individual’s total probability of infection at time *t* is calculated as $[\sum_{j} 1-\left( 1-p_{ij}^{S} \right)^{N^{S}}+\sum_{j} 1-\left( 1-p_{ij}^{I} \right)^{N_{ij}^{I}}]$. An uninfected individual can only be infected once in any time step. Upon infection with HIV, an individual is marked as HIV-positive and enters the acute phase. The individual’s assigned viral load and CD4 count, initiated at the time of that person’s entrance into the model, are now “active.” Upon infection with HCV, an individual is marked as HCV-positive and enters the acute phase. The infection is randomly assigned a genotype [15]. We assumed that an individual cannot begin an infection either aware or on treatment.

Table H provides parameter values and data sources related to non-specific disease transmission. Table I provides parameter values and data sources related to HIV- and HCV-specific disease transmission.

## *Disease Progression*

In keeping with the level of detail appropriate to capture substantial differences in infectivity, quality of life, and treatment costs, we tracked continuous viral load and CD4 count variables for HIV and we modeled HCV as a Markov process with discrete disease stages. At each monthly time step, we updated these disease-specific parameters for each individual already infected at the beginning of the month.

### HIV Progression

HIV infection begins with a brief and highly infectious acute phase [6]. Antiretroviral treatment (ART) slows HIV progression [44], which is characterized by increasing viral load and decreasing CD4 count, both of which are modeled as continuous variables [8, 44]. Opportunistic infections, which decrease quality of life and increase mortality risk, are more likely to occur at lower CD4 counts [44]. In order to enroll in ART, individuals must first become aware of their infectious status, which occurs via testing [6, 26].

If an individual has been HIV-infected for more than three months [6], that person transitions out of the acute phase. If the individual did not begin the month in the acute phase, he or she may develop an opportunistic infection with a probability that depends on CD4 count [44]. The opportunistic infection will lower the person’s CD4 count [44]. In the absence of treatment, the person experiences a – possibly additional – CD4 drop drawn from a viral-load-dependent distribution (the drop may potentially be negative; that is, a small gain is also possible) [45]. With effective treatment (i.e., treatment with a sufficiently high adherence level), viral load decreases at a fixed increment for the first six months, scaled by adherence level [44, 46]. We assumed that viral load will not drop past a set point of 1.7 log_10_ copies/ml [44], where 2.3 log_10_ copies/ml is considered virally suppressed [2]. We assumed that viral load remains constant after the first 6 months [44]. If an opportunistic infection did not occur in a given month, then an individual on effective treatment for less than 48 months will experience a CD4 rise that diminishes with the duration of treatment [44] and is scaled by adherence level [6]. The CD4 increase is also age-dependent, with individuals over age 40 experiencing less gain [44].

Table J provides parameter values and data sources related to HIV progression.

### HCV Progression

HCV progression is modeled as a Markov process between stages: a noninfectious, acute phase, followed by an infectious, chronic phase that progresses through liver fibrosis stages F0, F1, F2, F3, F4, and finally end-stage liver disease [9, 13, 47]. The progression rate is affected by sex, age, HIV status, and HIV treatment status [13]. Quality of life decreases throughout the progression [9]. In the acute and F0 stages, an individual can spontaneously clear HCV infection [9, 48]; afterwards, treatment is required to clear the infection [9, 14].

If an individual has acute HCV infection for more than 6 months [47], the person may either clear the infection or move into the F0 stage of chronic infection. The clearing probability is lower if the individual is co-infected with HIV [9, 48]. Because no additional costs or QALY losses are associated with acute HCV infection in the model, we only check whether the individual has cleared the infection once, at the end of the 6-month period. (We note that, if individuals clear HCV in less than 6 months, they would be re-eligible to be infected at a sooner date, making the pool of uninfected individuals higher than under our assumption, but this is unlikely to have a substantial impact on our calculations.)

If an individual begins the month with chronic HCV infection and is on treatment and not in the ESLD stage of HCV, he or she is eligible to clear the infection at the end of the treatment period. The probability that an individual clears the infection is based on the person’s race (black or not), the stage of infection (clearing probability decreasing with severity of stage), whether the person is co-infected with HIV (clearing probability with HIV co-infection is lower), and whether the person is CC-type or non-CC-type (CC-type individuals are better able to clear the infection) [9, 14].

If the infected individual did not clear HCV with treatment and is in the F0 stage, the person may still spontaneously clear the infection. The probability of spontaneous clearance is lower if the person is co-infected with HIV [9, 48]. If the individual does not clear the HCV infection in the current month, the infection will progress to the next stage (unless it is in the final ESLD stage), with a probability that is sex- and age-dependent (progression rates are highest for older males) and higher with HIV co-infection, although the effects of HIV can be reduced if the individual is on effective ART [13].

Table K provides parameter values and data sources related to HCV progression.

## *Disease Awareness and Treatment*

Individuals must be aware of their infection to initiate treatment. For instance, in the case of HIV, individuals who begin the month unaware of their HIV infection are eligible to be screened, and individuals who begin the month aware but not on treatment are eligible to enroll in ART. We assumed that individuals do not receive HIV or HCV care while in the incarceration system, nor are they screened at time of entry. It is unclear whether HIV and HCV care are provided consistently within jails and/or prisons and, if provided, what quality the care has. For this reason, we make the assumption that care would be sporadic at best and so do not consider effects from potential HIV and HCV care while incarcerated. Table L provides parameter values and data sources related to disease awareness and treatment.

### HIV Awareness

HIV-unaware individuals are tested at a probability that is dependent upon their risk group and community program enrollment. PWID in NSP are more likely to be tested for HIV than PWID not in NSP [6]. Similarly, PWID and PWUD receiving SUDT are more likely to be tested for HIV than PWID and PWUD not receiving SUDT [6]. Given the case management component of programs such as Seattle’s Law Enforcement Assisted Diversion Program (LEAD), we assume that individuals enrolled in the program have additionally increased chances of testing [49].

### HIV Treatment

HIV-aware individuals initiate and quit treatment at probabilities that are dependent upon their risk group and community program enrollment. PWID and PWUD receiving SUDT are more likely to begin HIV treatment than those who are not enrolled [6]. Conversely, quitting rates are lower for PWID and PWUD receiving SUDT than for those not receiving SUDT [6]. We assume that, as a consequence of the case management component of a diversion program, individuals enrolled in the program have additionally increased chances of starting ART and lowered chances of quitting [49].

At the time of incarceration, we assumed that individuals cease HIV treatment if they are on it. Because the majority of misdemeanor jail stays are one week or less and the average stay is 21 days [21], and because PWID and PWUD frequently cycle in and out of the incarceration system, if, at the time of ART initiation, an individual has ceased HIV treatment less than a month ago, we did not restart that person’s months-on-treatment count; that is, we avoided artificially inflating the viral load decrease and CD4 increase that could otherwise occur in the model by constantly quitting and restarting treatment.

Because PWID and PWUD have higher ART quit rates induced by higher incarceration rates, to keep enrollment calibrated to reasonably constant or increasing levels, we assumed that PWID and PWUD also have a higher probability of initiating ART than low-risk individuals. Out of context these numbers can appear misleading.

### HCV Treatment

We did not separately include a marker for HCV awareness nor did we explicitly model HCV testing. Because we assumed that the probability of initiating HCV treatment is very low [11, 12], the simplification does not create the risk of artificially inflating HCV treatment levels in the population.

As with HIV, HCV treatment initiation depends on risk group and enrollment in community programs. In practice, we modeled these processes identically, with a multiplier less than 1 that scales entry probabilities for HCV treatment. Quitting HCV treatment depends on the individual’s adherence levels (see *HCV, Initial Conditions*).

We assumed that at the time an individual progresses to ESLD, the person will cease HCV treatment if on it, as at this point there is no benefit from treatment, and only high costs and QALY losses from continuing (12). This drop is automatically triggered in the model. (In reality, the discrete distinctions between stages are a simplification, and treatment would not necessarily halt at such an arbitrary juncture. However, the simplification is unlikely to affect our calculations.)

At the time of incarceration, we assumed that individuals cease HCV treatment if they are on it. Because HCV treatment is expensive (12), has QALY decrements (12), and quit rates are already high, we assumed that even if the jail stay is short, treatment is not resumed upon release, as exit from the incarceration system can severely disrupt daily life.

## *Community Programs*

Table M provides parameter values and data sources related to community program transitions.

### NSP and SUDT

We assumed that enrollments in community programs are correlated: thus, a PWID using NSP is more likely to engage with SUDT and an individual connected to the diversion program is more likely to join both NSP and SUDT and less likely to quit these programs. At the time of incarceration, an individual ceases connection with both NSP and SUDT and does not automatically reconnect once released. This makes sense for SUDT especially, as there are often long waiting lists for SUDT and individuals may have to surrender their place. Opioid agonist therapies and rehab programs are sporadically offered within the incarceration system; we made the assumption that these are not available or not available at an effective level [34, 50]. As we did not model disease transmission within jails or prisons, this assumption only has impact on individual trajectories and quality of life. Since entry into the incarceration system can severely disrupt daily life, we also assumed that housing and other anchoring habits might change at the time of incarceration, making it not unreasonable for it to take a period of time to re-establish NSP use. We calibrated entry and quitting rates for NSP and SUDT such that the additional quit rates from incarceration are balanced and the percent of PWID and PWUD enrolled over time is constant (see Model Calibration).

While NSP enrollment is binary (an individual is either enrolled or not), we modeled SUDT with a waiting list since many individuals with interest in receiving opioid agonist therapy or enrolling in rehab programs never make contact with that service [51, 52]. For a PWID or PWUD who is not enrolled in SUDT nor on a waiting list for it, we assumed that the individual seeks out SUDT with a probability that is dependent on their risk group, NSP status, and diversion program status. Given that a suitable random number is drawn, another random number determines whether they immediately join SUDT (i.e., their waiting time is less than 1 month) or whether they join a waiting list and remain on the list for at least 1 month [51, 52]. A fraction of individuals seeking SUDT will withdraw from the waiting list and be lost before the next time step [51, 52]. Individuals still on the waiting list at the start of the month can either enter SUDT, quit the waiting list, or remain on the list for another month. Those enrolled in SUDT may quit at any time. We assume that quitting is less likely for individuals enrolled in the diversion program because of that program’s case management component [49].

### Diversion Program

The manuscript describes how we modeled the diversion program, with additional details in the *Criminal Activity and Incarceration* section of this supplement. Table N provides parameter values and data sources related to the model implementation of such a program.

## *Demographic Transitions*

At every time step, new individuals enter the model. The number of new entrants is determined such that the population remains largely constant with slight upward trend over 10 years [53]. New individuals are aged 18, are low risk, and are HIV- and HCV-negative. Their race, gender, and sex/sexual orientation are determined in keeping with current demographics in King County, Washington [2, 53-56].

Low-risk individuals may initiate drug use and become PWUD or PWID [56]. There is a separate probability for joining each risk group, calibrated to maintain risk group proportions over 5 years. These probabilities are higher for individuals under age 30 [56]. Most transitions into the PWID population are from PWUD, whose transition probabilities we assumed to be age-independent [57, 58]. PWID and PWUD may also quit drug use, either through a drug court program (which affects a very small number) [29] or through SUDT [27].

Table O provides parameter values and data sources related to demographic transitions.

## *Mortality*

Background mortality rates by age, sex, and race were taken from US life tables [59]. We assumed no direct increase in the background mortality rate due to an individual’s risk group, but PWID and PWUD have monthly probabilities of overdose [31, 36, 60-62], moderated by SUDT enrollment [27, 32, 62], and PWID have an additional probability of overdose at the time of release from the incarceration system [33, 62]. Overdose deaths are tracked separately. HIV infection increases mortality via CD4 count and opportunistic infections, but CD4 count-related mortality is moderated by being on effective ART [44, 45]. Across the F0-F4 stages, chronic HCV increases mortality by an amount that depends on both race and sex but is otherwise constant [15]. HCV-infected individuals with ESLD have a much higher probability of death from liver disease than individuals in stages F0-F4 [15]. We note that mortality rates are indirectly raised for PWID and PWUD because HIV and HCV prevalence are much higher in these populations than in the low-risk population. Table P provides parameter values and data sources related to mortality.

# Model Calibration

A majority of input parameters were calculated explicitly from published King County, Washington data or were well established in the literature on disease natural history and other infectious disease models. A subset of parameters had unknown or wide uncertainty intervals, and it was these parameters alone that we varied in a calibration process. For tractability, we took a simple, incremental approach. We manually varied subsets of model parameters to stabilize subsets of the model dynamics.

We undertook the following process. We assumed that incarceration dynamics did not depend on infection dynamics. Therefore we could isolate the incarceration dynamics and, turning off other aspects of the model, adjust incarceration-related parameters to replicate constant crime rates and jail and prison populations (Figures A-D). For this subset of model inputs, we performed a simplified version of calibration described in [27, 63], drawing from uniform ranges instead of parameterized distributions. We performed 15 “starter” simulations with random draws from conceivable ranges for each parameter and observed model output on key demographic targets. In large-scale model calibrations, this process would be automated and formalized, with each set of parameters scored using an objective function. Instead, given the scope of this calibration and the relative certainty of most parameters, we performed the calibration manually, although, in practice, we were approximating the same approach. The “starter” simulations provided intuition into how model dynamics depended on input values, and we narrowed our ranges accordingly, running another 15 simulations iteratively until the ranges were sufficiently tight that we could identify a reasonable set of parameters that reproduced demographic data in King County, Washington.

Next, having established incarceration-related inputs, we used a similar approach to calibrate community program enrollments (Figures E and F show results for the PWID population), which depended on incarceration rates but we assumed could still be isolated from disease dynamics. Disease incidence, awareness, and treatment all depended upon community program enrollments, so once we had adjusted those parameters appropriately we could calibrate to the remaining epidemiologic targets (Figures G-J show results for the PWID population). We began with HIV-related parameters, which involved interplay between risk groups, and, having established those, adjusted HCV-related parameters, which were mostly internal to the PWID community.

In the case of HIV incidence, we calibrated to historic King County, Washington data both in terms of absolute numbers (which were relatively stable over the past 5 years) and in terms of the distribution over risk, sex, and sexual orientation groups (which were presented as averages over the past 5 years) [2]. For treatment and awareness, given that both were high in all populations, we calibrated to current levels and aimed for constant levels or slight upward trending [2]. HCV targets were harder to identify. We estimated current infection rates given national prevalence and infection rates and the relative prevalence of HCV in King County, Washington [2-4, 10]. We also assumed that, given the higher prevalence of HCV in older generations, HCV prevalence would decrease over time in all risk groups [2-4, 10].

We calibrated primarily to the following targets: PWID population (proportion of total population), PWID NSP population (proportion of PWID population), PWID SUDT population (proportion of PWID population), PWID overdose deaths (monthly), PWID HIV prevalence, PWID HIV awareness (proportion of HIV-positive PWID population), PWID on HIV treatment (proportion of HIV-positive PWID population), PWID HCV prevalence, PWUD population, PWUD SUDT population, PWUD overdose deaths, PWUD HIV prevalence, PWUD HIV awareness, PWUD on HIV treatment, PWUD HCV prevalence, MSM population, MSM HIV prevalence, MSM HIV awareness, MSM on HIV treatment, low-risk HIV prevalence, low-risk HIV awareness, low-risk HIV on HIV treatment, low-risk HCV prevalence, total population size, total HIV incidence (monthly), PWID HIV incidence (proportion of total incidence), PWID/MSM HIV incidence (proportion of total incidence), MSM HIV incidence (proportion of total incidence), low-risk HIV incidence (proportion of total incidence), total HCV incidence (monthly), HIV diagnosis (monthly), HIV-related mortality (monthly), misdemeanor arrests (monthly), felony arrests (monthly), jail population (monthly), prison population (monthly).

# Economic Model

Every state in the model has an associated monthly QALY value. This value is a function of an age-, sex-, and risk group-dependent QALY baseline [64]; multipliers that are greater or less than 1 that raise or lower quality of life (e.g., SUDT enrollment raises quality of life, while HIV infection lowers quality of life); and possible decrements from HCV treatment [9]. HIV quality-of-life multipliers are based on CD4 count unless an individual develops an opportunistic infection, in which case the usual CD4 count QALY multiplier is replaced by a lower multiplier for that month [65]. We did not include a quality-of-life multiplier specifically for ART, as the effect of ART is reflected in an individual’s CD4 count. HCV-infected individuals may continue to have QALY multipliers less than 1 even after a sustained virologic response from HCV treatment, depending on the stage from which they recovered [9].

Because there is scant supporting data, we conservatively assumed no QALY decrement associated with incarceration. While it is likely that entering the incarceration system generates substantial QALY losses, some sources suggest that basic healthcare within the system can in fact improve quality of life for the very poor and marginalized who would not otherwise receive such services [50]. Therefore, our base case analysis assumed no direct change in quality of life due to incarceration, only the indirect change that comes from losing SUDT or treatment services.

Costs are associated with all states as well as some transitions. Any identified crime is associated with a one-time police response cost and a jail-booking fee, as well as a one-time misdemeanor or felony charge that covers legal proceedings [21]. HIV diagnosis also has a one-time cost [27]. Background healthcare usage is age-dependent, with individuals over 60 having substantially higher costs [27, 66], unless an individual is in prison, in which case a constant cost, which includes healthcare, is applied [21, 67]. We also included costs associated with HCV stage or sustained virologic response from later HCV stages and HCV treatment; with HIV CD4 count, opportunistic infections, and ART; with NSP, SUDT, and the diversion program; and with jail.

Table Q provides parameter values and data sources related to costs and QALYs.

We discounted all costs and QALYs at a 3% annual rate [68, 69]. Additionally, we calculated lifetime costs and QALYs by age, risk group, and infection status (infected with both HIV and HCV, either HIV or HCV, or neither). We took a dot product of a vector of population size at the end of the model run with a vector of lifetime costs (discounted out at 10 years), and another of lifetime benefits (also discounted), to determine the total lifetime costs and QALYs for the population.

We briefly summarize how we calculated lifetime costs; the QALY process is identical. First, for each risk and infection group, we began with 200,000 individuals and ran the simulation in the absence of infection or population entry for the average-aged individual in the age 60-74 group until they matured out of the model at 74. We stored the cumulative costs, then performed the same simulation for the average-aged individual in the age 50-59 group until they reached the age of 60 and stored the costs again. We also stored the probability of surviving till the end of the time horizon, $p_{50-59}$. After performing these simulations for each age group, we then calculated lifetime costs. For instance, for an individual in the 40-49 age group, $C=\frac{C_{40-49}}{1}+p_{40-49}\frac{C_{50-59}}{{1.03}^{5}}+p_{50-59}\frac{C_{60-74}}{({1.03}^{5})({1.03}^{5})}$. This approach reduces run time; similar calculations have been used for other microsimulations [70].

**Figure A. Calibrated misdemeanor arrests**


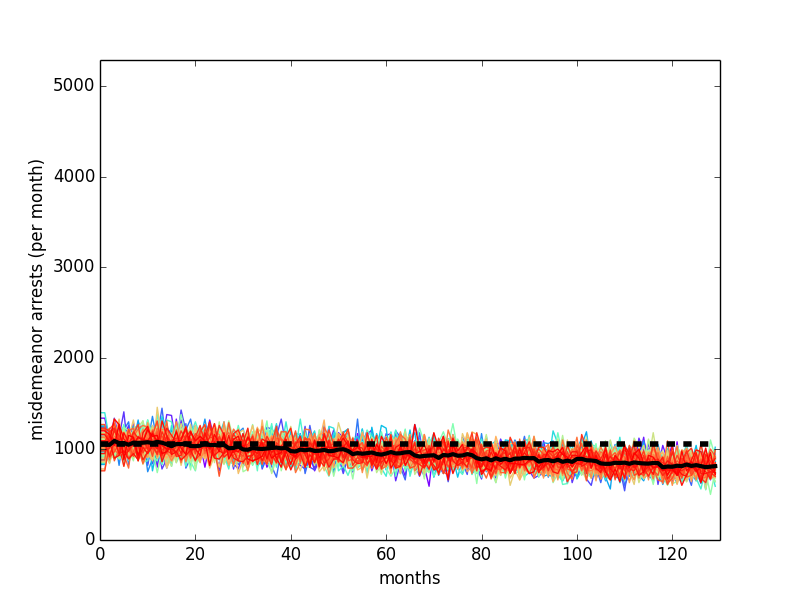


Colored lines plots sampled trial runs under the model’s status quo. The solid black line plots the average among sampled trial runs. The dotted black line plots the current demographic estimate in King County, Washington.

**Figure B. Calibrated jail population**

**
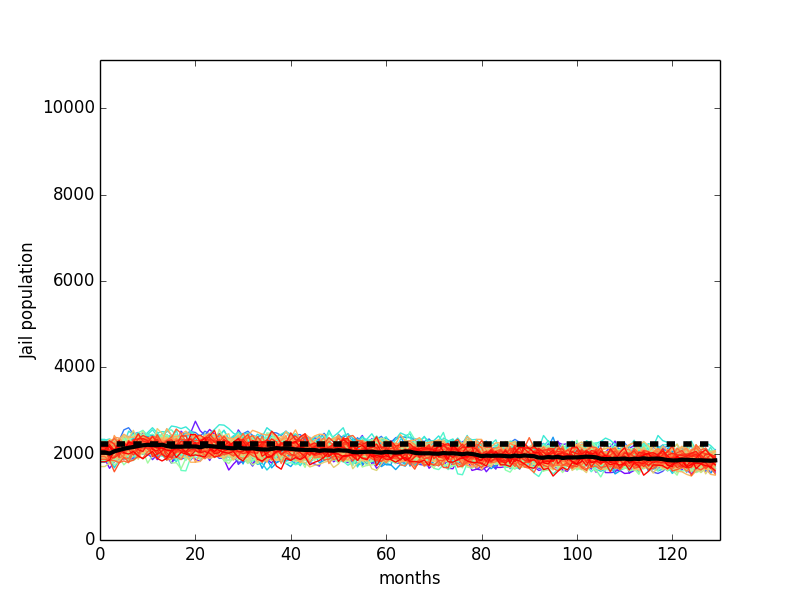
**

Colored lines plots sampled trial runs under the model’s status quo. The solid black line plots the average among sampled trial runs. The dotted black line plots the current demographic estimate in King County, Washington.

**Figure C. Calibrated felony arrests**

**
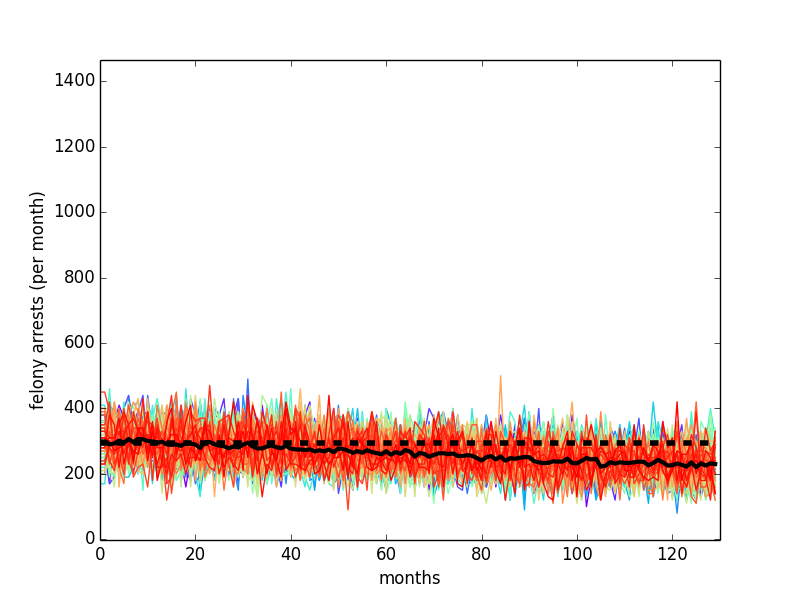
**

Colored lines plots sampled trial runs under the model’s status quo. The solid black line plots the average among sampled trial runs. The dotted black line plots the current demographic estimate in King County, Washington.

**Figure D. Calibrated prison population**


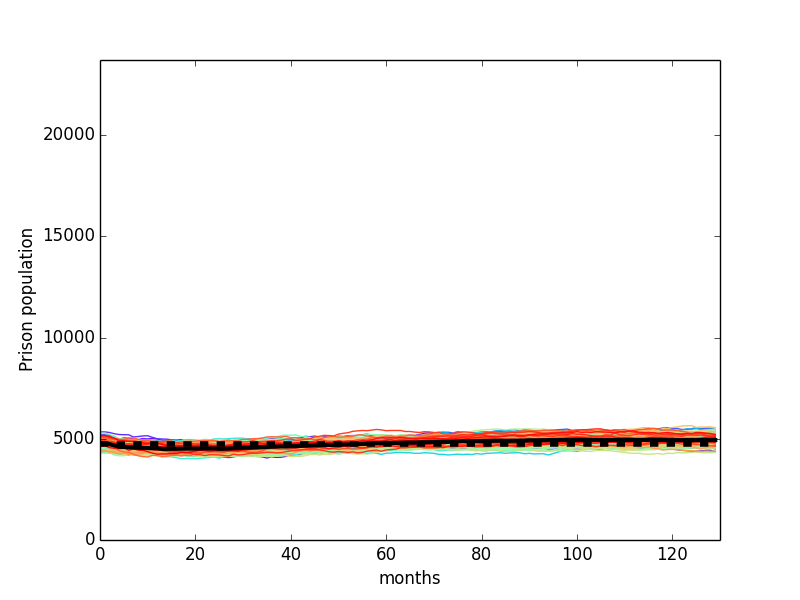


Colored lines plots sampled trial runs under the model’s status quo. The solid black line plots the average among sampled trial runs. The dotted black line plots the current demographic estimate in King County, Washington.

**Figure E. Calibrated NSP population, PWID**


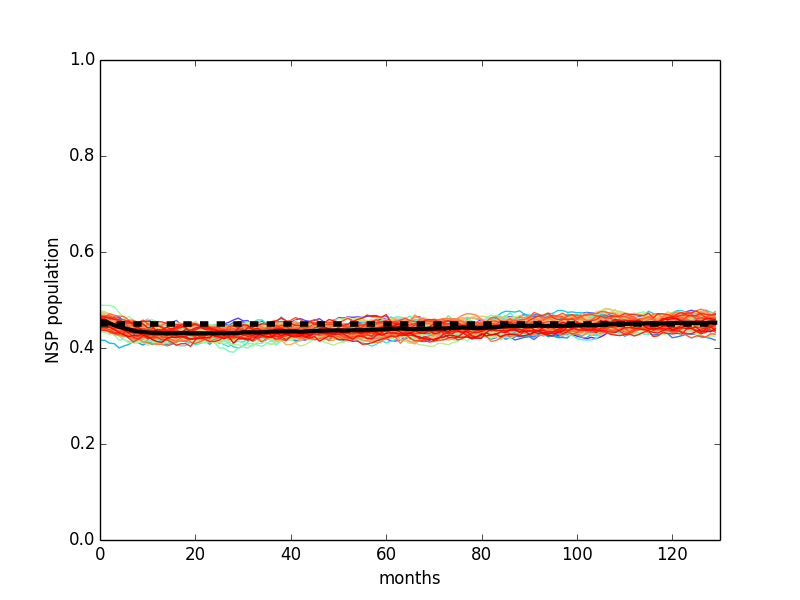


Colored lines plots sampled trial runs under the model’s status quo. The solid black line plots the average among sampled trial runs. The dotted black line plots the current demographic estimate in King County, Washington.

**Figure F. Calibrated SUDT population, PWID**


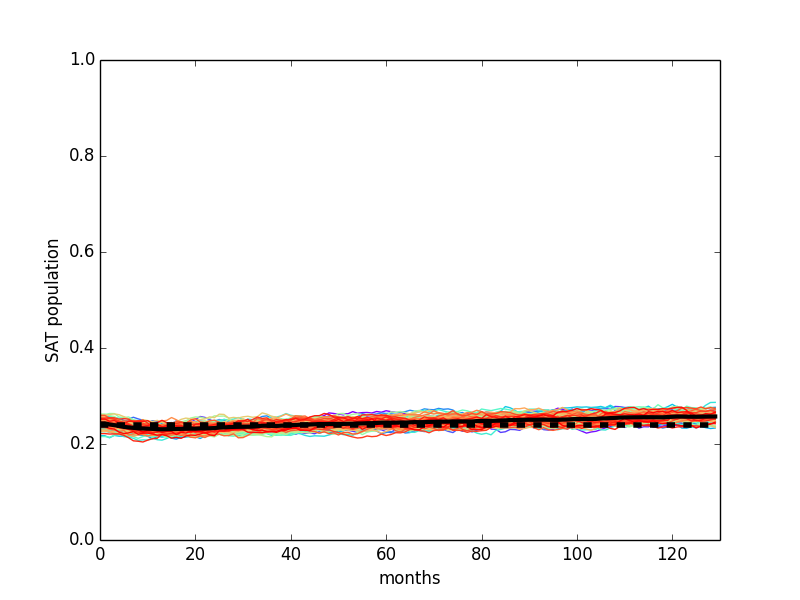


Colored lines plots sampled trial runs under the model’s status quo. The solid black line plots the average among sampled trial runs. The dotted black line plots the current demographic estimate in King County, Washington.

**Figure G. Calibrated HIV prevalence, PWID**


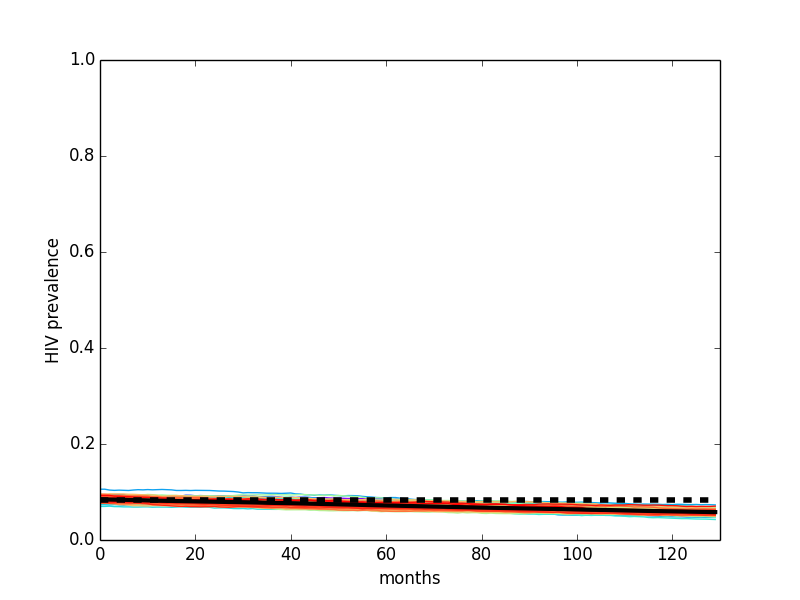


Colored lines plots sampled trial runs under the model’s status quo. The solid black line plots the average among sampled trial runs. The dotted black line plots the current demographic estimate in King County, Washington.

**Figure H. Calibrated HIV awareness, PWID**


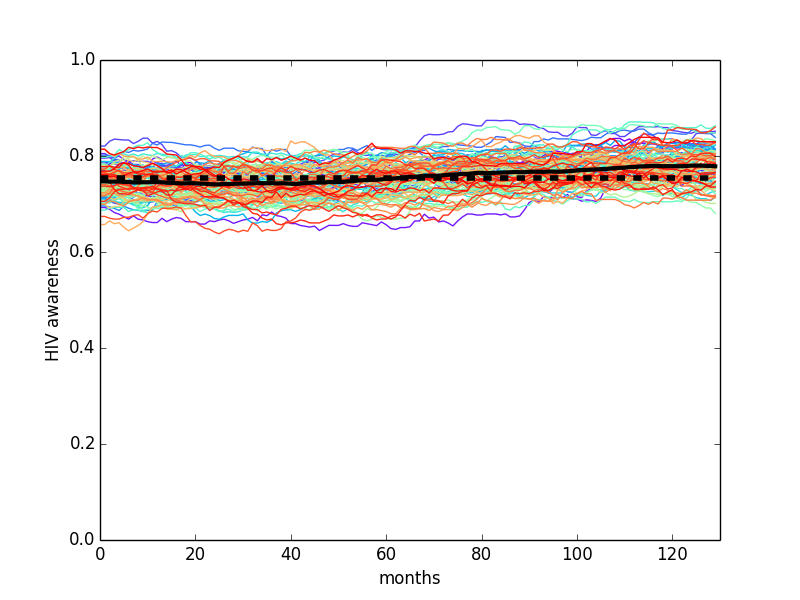


Colored lines plots sampled trial runs under the model’s status quo. The solid black line plots the average among sampled trial runs. The dotted black line plots the current demographic estimate in King County, Washington.

**Figure I. Calibrated HIV treatment, PWID**


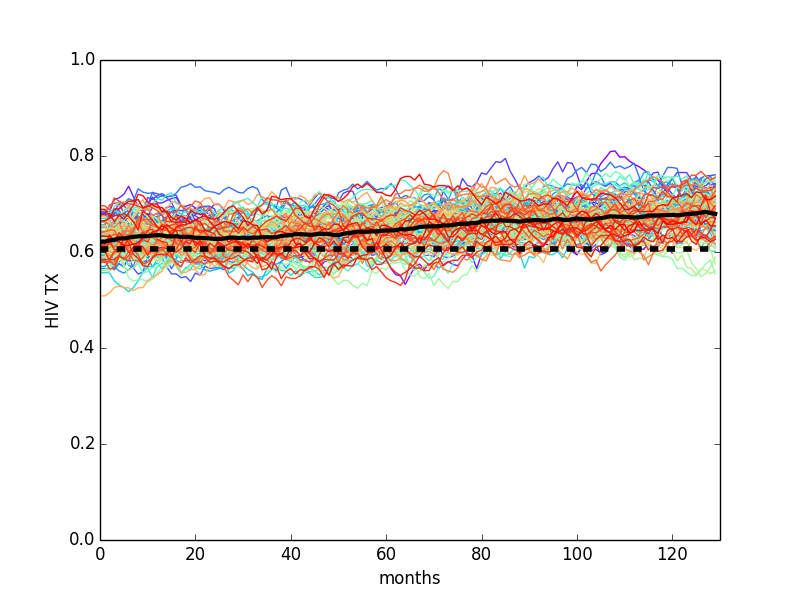


Colored lines plots sampled trial runs under the model’s status quo. The solid black line plots the average among sampled trial runs. The dotted black line plots the current demographic estimate in King County, Washington.

**Figure J. Calibrated HCV prevalence, PWID**


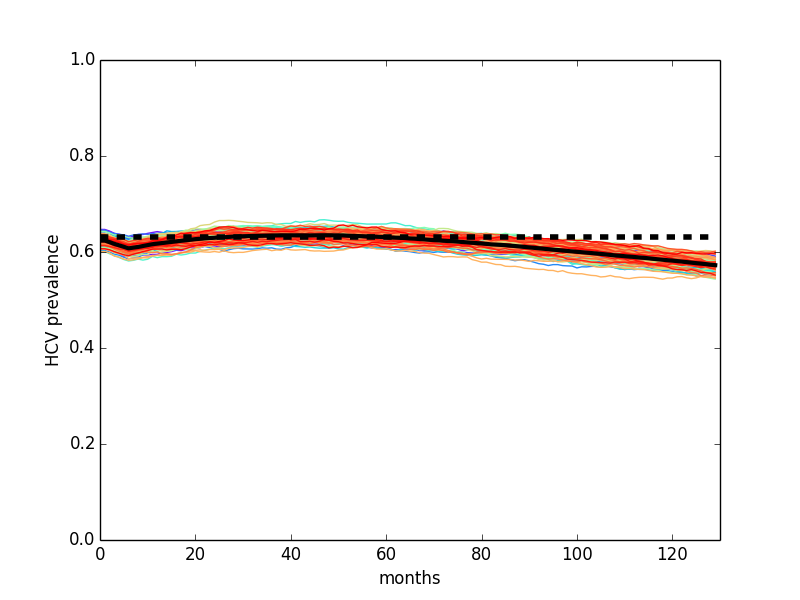


Colored lines plots sampled trial runs under the model’s status quo. The solid black line plots the average among sampled trial runs. The dotted black line plots the current demographic estimate in King County, Washington.

**Figure K. Cost-Efficient Frontier (Base Case)**


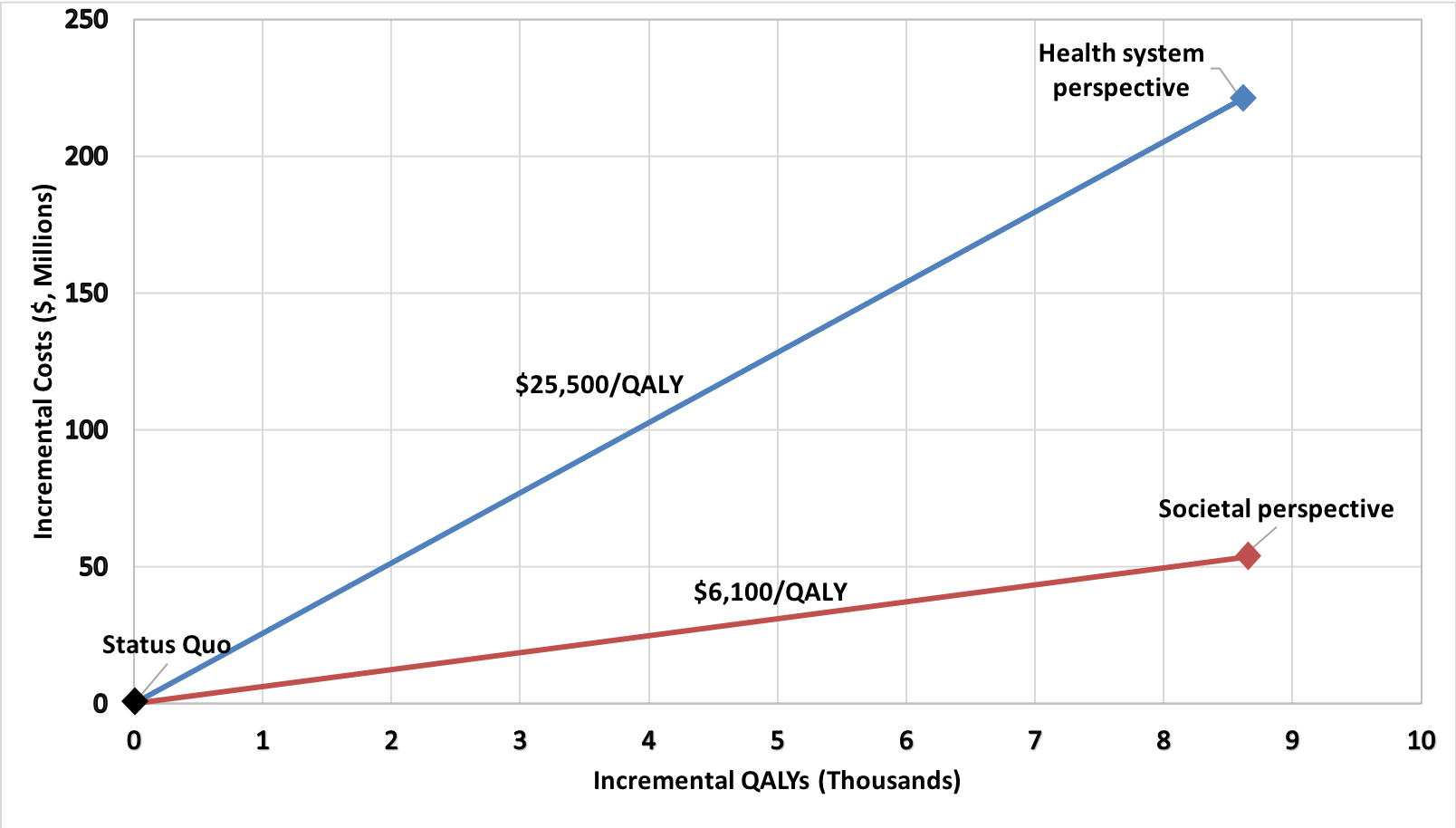


When considering healthcare costs only (both inside and outside the criminal justice system), the program cost $25,500 per QALY gained. Including additional savings from reduced incarceration (societal perspective) improved the ICER to $6,200 per QALY gained.

**Table A. Model Demographics^[[1]](#footnote-2)^**

| **Parameter** | **Value** | **Source** |
| --- | --- | --- |
| King County population, ages 18-74 | 1,390,302 | [54] |
| **PWID** |  |  |
| % of population | 0.0165 | [2, 54, 56] |
| **Race and sex** |  | [2] |
| % white | 0.57 |  |
| % black | 0.23 |  |
| % other | 0.20 |  |
| % female | 0.33 |  |
| **Age (HIV uninfected)** |  | [2, 53] |
| 18-29 | 0.155 |  |
| 30-39 | 0.246 |  |
| 40-49 | 0.259 |  |
| 50-59 | 0.255 |  |
| 60-74 | 0.085 |  |
| **MSM (HIV uninfected)** |  | [2, 55] |
| P(MSM\|white male) | 0.083 |  |
| P(MSM\|black male) | 0.046 |  |
| P(MSM\|other male) | 0.099 |  |
| **Non-PWID** |  |  |
| **Race and sex** |  | [2, 54, 56] |
| % white | 0.702 |  |
| % black | 0.064 |  |
| % other | 0.234 |  |
| % female | 0.503 |  |
| **Age (HIV uninfected, non-MSM)** |  | [2, 53] |
| 18-29 | 0.233 |  |
| 30-39 | 0.214 |  |
| 40-49 | 0.209 |  |
| 50-59 | 0.191 |  |
| 60-74 | 0.154 |  |
| **MSM (HIV uninfected)** |  | [2, 55] |
| P(MSM\|white male) | 0.038 |  |
| P(MSM\|black male) | 0.089 |  |
| P(MSM\|other male) | 0.046 |  |
| **Age (HIV-uninfected MSM)** |  | [2, 53] |
| 18-29 | 0.372 |  |
| 30-39 | 0.277 |  |
| 40-49 | 0.182 |  |
| 50-59 | 0.118 |  |
| 60-74 | 0.051 |  |
| **PWUD (HIV uninfected)** |  | [2, 6, 55] |
| **Heterosexual female** |  |  |
| P(PWUD \|white non-PWID) | 0.039 |  |
| P(PWUD \|black non-PWID) | 0.173 |  |
| P(PWUD \|other non-PWID) | 0.041 |  |
| **Heterosexual male** |  |  |
| P(PWUD \|white non-PWID) | 0.051 |  |
| P(PWUD \|black non-PWID) | 0.241 |  |
| P(PWUD \|other non-PWID) | 0.054 |  |
| **MSM** |  |  |
| P(PWUD \|white non-PWID) | 0.177 |  |
| P(PWUD \|black non-PWID) | 0.179 |  |
| P(PWUD \|other non-PWID) | 0.171 |  |

**Table B. HIV Demographics^[[2]](#footnote-3)^**

| **Parameter** | **Value** | **Source** |
| --- | --- | --- |
| **PWID** |  |  |
| **HIV Prevalence** |  | [2] |
| **Female** |  |  |
| P(HIV\|white) | 0.049 |  |
| P(HIV\|black) | 0.071 |  |
| P(HIV\|other) | 0.076 |  |
| **Male** |  |  |
| P(HIV\|white) | 0.080 |  |
| P(HIV\|black) | 0.114 |  |
| P(HIV\|other) | 0.123 |  |
| **Age distribution (HIV infected)** |  | [2, 53] |
| 18-29 | 0.097 |  |
| 30-39 | 0.290 |  |
| 40-49 | 0.387 |  |
| 50-59 | 0.170 |  |
| 60-74 | 0.0565 |  |
| **Awareness of HIV status** |  |  |
| Fraction aware | 0.755 | [7] |
| P(AIDS\|aware female) | 0.637 | [2] |
| P(AIDS\|aware heterosexual male) | 0.696 | [2] |
| P(AIDS\|aware MSM) | 0.614 | [2] |
| **HIV Treatment** |  |  |
| P(ART\|aware) | 0.802 | [2] |
| Fraction achieving >90% adherence | 0.810 | [6] |
| **MSM** |  | [2, 55] |
| P(MSM\|HIV infected, white male) | 0.962 |  |
| P(MSM\|HIV infected, black male) | 0.357 |  |
| P(MSM\|HIV infected, other male) | 0.710 |  |
| **Non-PWID** |  |  |
| **HIV Prevalence** |  | [2, 54, 56] |
| **Female** |  |  |
| P(HIV\|white) | 0.00082 |  |
| P(HIV\|black) | 0.00231 |  |
| P(HIV\|other) | 0.00071 |  |
| **Male** |  |  |
| P(HIV\|white) | 0.00857 |  |
| P(HIV\|black) | 0.0241 |  |
| P(HIV\|other) | 0.00735 |  |
| **Age distribution (HIV infected)** |  | [2, 53] |
| 18-29 | 0.088 |  |
| 30-39 | 0.149 |  |
| 40-49 | 0.250 |  |
| 50-59 | 0.350 |  |
| 60-74 | 0.163 |  |
| **Awareness of HIV status** |  | [2] |
| Fraction aware | 0.92 |  |
| P(AIDS\|aware female) | 0.569 |  |
| P(AIDS\|aware heterosexual male) | 0.706 |  |
| **HIV Treatment** |  |  |
| P(ART\|aware) | 0.880 | [2] |
| Fraction achieving >90% adherence | 0.818 | [6] |
| **MSM** |  |  |
| P(MSM\|HIV infected, white male) | 0.828 | [2, 55] |
| P(MSM\|HIV infected, black male) | 0.721 | [2, 55] |
| P(MSM\|HIV infected, other male) | 0.965 | [2, 55] |
| **Age distribution (HIV infected)** |  | [2, 53] |
| 18-29 | 0.181 |  |
| 30-39 | 0.304 |  |
| 40-49 | 0.249 |  |
| 50-59 | 0.184 |  |
| 60-74 | 0.082 |  |
| **Awareness of HIV status** |  | [2] |
| Fraction aware | 0.920 |  |
| P(AIDS\|aware) | 0.507 |  |
| **HIV Treatment** |  |  |
| P(ART\|aware) | 0.902 | [2] |
| Fraction achieving >90% adherence | 0.820 | [6] |
| **PWUD^[[3]](#footnote-4)^** |  | [2, 6, 55] |
| **Heterosexual female** |  |  |
| P(PWUD \|white non-PWID) | 0.282 |  |
| P(PWUD \|black non-PWID) | 0.446 |  |
| P(PWUD \|other non-PWID) | 0.080 |  |
| **Heterosexual male** |  |  |
| P(PWUD \|white non-PWID) | 0.195 |  |
| P(PWUD \|black non-PWID) | 0.191 |  |
| P(PWUD \|other non-PWID) | 0.274 |  |
| **MSM** |  |  |
| P(PWUD \|white non-PWID) | 0.733 |  |
| P(PWUD \|black non-PWID) | 0.705 |  |
| P(PWUD \|other non-PWID) | 0.878 |  |
| **Untreated HIV** |  | [8] |
| **CD4 count distribution** |  |  |
| 0-50 | 0.104 |  |
| 51-200 | 0.272 |  |
| 201-350 | 0.261 |  |
| 351-500 | 0.177 |  |
| 500-700 | 0.186 |  |
| **Viral load (log_10_ copies/ml)** |  |  |
| Normal distribution, mean | 4.02 |  |
| Normal distribution, SD | 0.85 |  |
| Normal distribution, min | 2.60 |  |
| Normal distribution, max | 6.00 |  |
| **Viral load for virally suppressed HIV** |  | [2, 8] |
| Normal distribution, mean | 2.00 |  |
| Normal distribution, SD | 0.27 |  |
| Normal distribution, min | 0.10 |  |
| Normal distribution, max | 2.30 |  |

**Table C. HCV Demographics^[[4]](#footnote-5)^**

| **Parameter** | **Value** | **Source** |
| --- | --- | --- |
| **HCV prevalence, non-PWID/non-PWUD** |  | [3, 10] |
| **Female** |  |  |
| 18-29 | 0.00158 |  |
| 30-39 | 0.00344 |  |
| 40-49 | 0.00352 |  |
| 50-59 | 0.00965 |  |
| 60-74 | 0.0240 |  |
| **Male** |  |  |
| 18-29 | 0.00273 |  |
| 30-39 | 0.00594 |  |
| 40-49 | 0.00606 |  |
| 50-59 | 0.0165 |  |
| 60-74 | 0.0412 |  |
| **HCV prevalence, PWID** |  | [2, 3, 5] |
| 18-29 | 0.450 |  |
| 30-39 | 0.534 |  |
| 40-49 | 0.637 |  |
| 50-59 | 0.783 |  |
| 60-74 | 0.783 |  |
| **HCV prevalence, PWUD** |  | [3, 10] |
| 18-29 | 0.090 |  |
| 30-39 | 0.107 |  |
| 40-49 | 0.127 |  |
| 50-59 | 0.157 |  |
| 60-74 | 0.157 |  |
| **HCV treatment and recovery** |  |  |
| Fraction of PWID on treatment | 0.044 | [11, 12] |
| Fraction of non-PWID on treatment | 0.055 | Estimated |
| Fraction of HCV infections that are G2/3 genotype (6 months of treatment) | 0.3 | [14] |
| Fraction of HCV infections that are G1/4 genotype (12 months of treatment) | 0.7 | [14] |
| P(CC-type\|white) | 0.370 | [15] |
| P(CC-type\|black) | 0.140 | [15] |

**Table D. Partnerships and Mixing^[[5]](#footnote-6)^**

| **Parameter** | **Value** | **Source** |
| --- | --- | --- |
| **Negative binomial distributions for monthly partnership formation (n, p)** |  | [17] |
| **PWID** |  |  |
| Female | (0.25,0.40) |  |
| Heterosexual male | (0.25,0.40) |  |
| MSM | (0.50,0.40) |  |
| **PWUD** |  |  |
| Female | (0.30,0.40) |  |
| Heterosexual male | (0.25,0.50) |  |
| MSM | (0.65,0.32) |  |
| **Non-PWID, non-PWUD** |  |  |
| Female | (0.17,0.40) |  |
| Heterosexual male | (0.083,0.75) |  |
| MSM | (0.50,0.50) |  |
| **Macro-network** |  | [6, 17] |
| Fraction of PWID partners who are chosen to be PWID | 0.80 |  |
| Fraction of PWID partners who are chosen to be PWUD | 0.18 |  |
| Fraction of PWUD partners who are chosen to be PWUD | 0.60 |  |
| Fraction of MSM partners who are chosen to be MSM | 0.85 |  |
| Fraction of non-PWID, non-PWUD partners who are chosen to be non-PWID, non-PWUD | 1.00 |  |
| Age selection | see tables below | [18] |
| **Characterizing partnerships** |  |  |
| Fraction of relationships that are casual | 0.342 | [19] |
| Fraction of PWID partners that are injecting only | 0.600 | [6] |

| **Male Sexual Partner Matrix)** | | Age | | | | | |
| --- | --- | --- | --- | --- | --- | --- | --- |
|  |  | 18-19 | 20-29 | 30-39 | 40-49 | 50-59 | 60-74 |
| Age | 18-19 | 0.973 | 0.020 | 0.00420 | 0.00140 | 0.00140 | 0.000 |
|  | 20-29 | 0.0850 | 0.800 | 0.100 | 0.00857 | 0.00429 | 0.00214 |
|  | 30-39 | 0.0560 | 0.224 | 0.630 | 0.060 | 0.020 | 0.010 |
|  | 40-49 | 0.0418 | 0.167 | 0.170 | 0.560 | 0.0520 | 0.00900 |
|  | 50-59 | 0.0276 | 0.110 | 0.179 | 0.17875 | 0.500 | 0.00450 |
|  | 60-74 | 0.0188 | 0.0375 | 0.0938 | 0.200 | 0.200 | 0.450 |

| **Female Sexual Partner Matrix** | | Age | | | | | |
| --- | --- | --- | --- | --- | --- | --- | --- |
|  |  | 18-19 | 20-29 | 30-39 | 40-49 | 50-59 | 60-74 |
| Age | 18-19 | 0.91 | 0.0780 | 0.00720 | 0.00240 | 0.00240 | 0.00 |
|  | 20-29 | 0.020 | 0.760 | 0.150 | 0.028 | 0.0280 | 0.0140 |
|  | 30-39 | 0.0040 | 0.0260 | 0.740 | 0.150 | 0.0533 | 0.0267 |
|  | 40-49 | 0.00460 | 0.0184 | 0.0820 | 0.690 | 0.140 | 0.0650 |
|  | 50-59 | 0.00243 | 0.00486 | 0.138 | 0.640 | 0.205 | 0.000 |
|  | 60-74 | 0.00 | 0.00486 | 0.0121 | 0.0197 | 0.118 | 0.845 |

**Table E. Incarceration, Initial Conditions^[[6]](#footnote-7)^**

| **Parameter** | **Value** | **Source** |
| --- | --- | --- |
| **Prison** |  |  |
| Fraction incarcerated in prison | 0.680 | [21, 23] |
| **Sentence distribution** |  | [24, 71] |
| 1-2 years | 0.221 |  |
| 2-5 years | 0.363 |  |
| 5-10 years | 0.312 |  |
| 10+ years | 0.105 |  |
| **Jail** |  |  |
| **Misdemeanor sentence distribution** |  | [21] |
| 1 week | 0.641 |  |
| 2 week | 0.103 |  |
| 3-4 week | 0.135 |  |
| 4-9 week | 0.055 |  |
| 9-13 week | 0.026 |  |
| 13-25 week | 0.026 |  |
| 26-52 week | 0.014 |  |
| **Pre-sentence felony** |  |  |
| Fraction of jail population | 0.578 | Estimated [21] |
| Uniformly distributed stay, weeks | [4,8] | Estimated [25] |
| **Felony sentence** |  | Estimated [21, 25] |
| Fraction of jail population | 0.109 |  |
| 2-week sentence | 0.11 |  |
| 3-4-week sentence | 0.70 |  |
| 4-9-week sentence | 0.18 |  |
| 9-13-week sentence | 0.01 |  |
| **Fraction currently incarcerated, by age** |  | [20-23] |
| **PWID** |  |  |
| **Female** |  |  |
| **White** |  |  |
| 18-29 | 0.085 |  |
| 30-39 | 0.043 |  |
| 40-49 | 0.027 |  |
| 50-59 | 0.016 |  |
| 60-74 | 0.017 |  |
| **Black** |  |  |
| 18-29 | 0.120 |  |
| 30-39 | 0.061 |  |
| 40-49 | 0.037 |  |
| 50-59 | 0.022 |  |
| 60-74 | 0.023 |  |
| **Other** |  |  |
| 18-29 | 0.035 |  |
| 30-39 | 0.018 |  |
| 40-49 | 0.012 |  |
| 50-59 | 0.006 |  |
| 60-74 | 0.007 |  |
| **Male** |  |  |
| **White** |  |  |
| 18-29 | 0.307 |  |
| 30-39 | 0.156 |  |
| 40-49 | 0.095 |  |
| 50-59 | 0.0567 |  |
| 60-74 | 0.060 |  |
| **Black** |  |  |
| 18-29 | 0.420 |  |
| 30-39 | 0.209 |  |
| 40-49 | 0.126 |  |
| 50-59 | 0.077 |  |
| 60-74 | 0.081 |  |
| **Other** |  |  |
| 18-29 | 0.126 |  |
| 30-39 | 0.062 |  |
| 40-49 | 0.038 |  |
| 50-59 | 0.023 |  |
| 60-74 | 0.024 |  |
| **Non-PWID, non-PWUD** |  |  |
| **Female** |  |  |
| **White** |  |  |
| 18-29 | 0.00034 |  |
| 30-39 | 0.00031 |  |
| 40-49 | 0.00021 |  |
| 50-59 | 0.00013 |  |
| 60-74 | 0.00005 |  |
| **Black** |  |  |
| 18-29 | 0.00413 |  |
| 30-39 | 0.00379 |  |
| 40-49 | 0.00255 |  |
| 50-59 | 0.00154 |  |
| 60-74 | 0.00067 |  |
| **Other** |  |  |
| 18-29 | 0.00027 |  |
| 30-39 | 0.00025 |  |
| 40-49 | 0.00017 |  |
| 50-59 | 0.00010 |  |
| 60-74 | 0.00004 |  |
| **Male** |  |  |
| **White** |  |  |
| 18-29 | 0.00366 |  |
| 30-39 | 0.00339 |  |
| 40-49 | 0.00232 |  |
| 50-59 | 0.00141 |  |
| 60-74 | 0.00062 |  |
| **Black** |  |  |
| 18-29 | 0.044 |  |
| 30-39 | 0.041 |  |
| 40-49 | 0.029 |  |
| 50-59 | 0.018 |  |
| 60-74 | 0.008 |  |
| **Other** |  |  |
| 18-29 | 0.00260 |  |
| 30-39 | 0.00242 |  |
| 40-49 | 0.00166 |  |
| 50-59 | 0.00101 |  |
| 60-74 | 0.00044 |  |
| **PWUD** |  |  |
| **Female** |  |  |
| **White** |  |  |
| 18-29 | 0.0188 |  |
| 30-39 | 0.0099 |  |
| 40-49 | 0.0062 |  |
| 50-59 | 0.0035 |  |
| 60-74 | 0.0037 |  |
| **Black** |  |  |
| 18-29 | 0.0263 |  |
| 30-39 | 0.0139 |  |
| 40-49 | 0.0087 |  |
| 50-59 | 0.0049 |  |
| 60-74 | 0.0051 |  |
| **Other** |  |  |
| 18-29 | 0.0076 |  |
| 30-39 | 0.0040 |  |
| 40-49 | 0.0025 |  |
| 50-59 | 0.0014 |  |
| 60-74 | 0.0015 |  |
| **Male** |  |  |
| **White** |  |  |
| 18-29 | 0.078 |  |
| 30-39 | 0.047 |  |
| 40-49 | 0.031 |  |
| 50-59 | 0.018 |  |
| 60-74 | 0.019 |  |
| **Black** |  |  |
| 18-29 | 0.123 |  |
| 30-39 | 0.070 |  |
| 40-49 | 0.046 |  |
| 50-59 | 0.026 |  |
| 60-74 | 0.027 |  |
| **Other** |  |  |
| 18-29 | 0.030 |  |
| 30-39 | 0.018 |  |
| 40-49 | 0.012 |  |
| 50-59 | 0.007 |  |
| 60-74 | 0.007 |  |

**Table F. SUDT and NSP Programs, Initial Conditions^[[7]](#footnote-8)^**

| **Parameter** | **Value** | **Source** |
| --- | --- | --- |
| **SUDT** |  |  |
| P(SUDT\|not on ART) | 0.211 | [2, 6, 56] |
| P(SUDT\|on ART) | 0.644 | [2, 6, 56] |
| Fraction of SUDT-associated individuals on waiting list | 0.018 | Estimated [51, 52, 72] |
| **NSP** |  |  |
| P(NSP\|on SUDT) | 0.850 | Estimated [6, 56] |
| P(NSP\|not on SUDT) | 0.324 | Estimated [6, 56] |

**Table G. Incarceration Transitions^[[8]](#footnote-9)^**

| **Parameter** | **Value** | **Source** |
| --- | --- | --- |
| **Chance of weekly identified crime** |  | [20, 21, 24, 30] |
| **PWID** |  |  |
| **Female** |  |  |
| **White** |  |  |
| 18-29 | 0.0037 |  |
| 30-39 | 0.0022 |  |
| 40-49 | 0.0013 |  |
| 50-59 | 0.0008 |  |
| 60-74 | 0.0008 |  |
| **Black** |  |  |
| 18-29 | 0.0093 |  |
| 30-39 | 0.0053 |  |
| 40-49 | 0.0033 |  |
| 50-59 | 0.0019 |  |
| 60-74 | 0.0020 |  |
| **Other** |  |  |
| 18-29 | 0.0107 |  |
| 30-39 | 0.0061 |  |
| 40-49 | 0.0037 |  |
| 50-59 | 0.0022 |  |
| 60-74 | 0.0023 |  |
| **Male** |  |  |
| **White** |  |  |
| 18-29 | 0.0149 |  |
| 30-39 | 0.0075 |  |
| 40-49 | 0.0046 |  |
| 50-59 | 0.0027 |  |
| 60-74 | 0.0029 |  |
| **Black** |  |  |
| 18-29 | 0.0373 |  |
| 30-39 | 0.0185 |  |
| 40-49 | 0.0112 |  |
| 50-59 | 0.0069 |  |
| 60-74 | 0.0072 |  |
| **Other** |  |  |
| 18-29 | 0.0430 |  |
| 30-39 | 0.0213 |  |
| 40-49 | 0.0128 |  |
| 50-59 | 0.0079 |  |
| 60-74 | 0.0083 |  |
| **PWUD** |  |  |
| **Female** |  |  |
| **White** |  |  |
| 18-29 | 0.0008 |  |
| 30-39 | 0.0005 |  |
| 40-49 | 0.0003 |  |
| 50-59 | 0.0002 |  |
| 60-74 | 0.0002 |  |
| **Black** |  |  |
| 18-29 | 0.0020 |  |
| 30-39 | 0.0012 |  |
| 40-49 | 0.0008 |  |
| 50-59 | 0.0004 |  |
| 60-74 | 0.0004 |  |
| **Other** |  |  |
| 18-29 | 0.0023 |  |
| 30-39 | 0.0014 |  |
| 40-49 | 0.0009 |  |
| 50-59 | 0.0005 |  |
| 60-74 | 0.0005 |  |
| **Male** |  |  |
| **White** |  |  |
| 18-29 | 0.0038 |  |
| 30-39 | 0.0023 |  |
| 40-49 | 0.0015 |  |
| 50-59 | 0.0009 |  |
| 60-74 | 0.0009 |  |
| **Black** |  |  |
| 18-29 | 0.0109 |  |
| 30-39 | 0.0062 |  |
| 40-49 | 0.0040 |  |
| 50-59 | 0.0023 |  |
| 60-74 | 0.0024 |  |
| **Other** |  |  |
| 18-29 | 0.0103 |  |
| 30-39 | 0.0062 |  |
| 40-49 | 0.0042 |  |
| 50-59 | 0.0025 |  |
| 60-74 | 0.0025 |  |
| **Community Programs** |  | [28] |
| Hazard ratio for criminal activity, SUDT | 0.914 |  |
| Hazard ratio for criminal activity, not on SUDT | 1.027 |  |
|  |  |  |
| **Felony** |  |  |
| Fraction of crimes that are felonies | 0.217 | [21] |
| Fraction of felonies resulting in prison sentence | 0.218 | [21, 24] |
| Fraction of felonies resulting in jail sentence | 0.430 | [21] |
| Fraction of felonies resulting in release after trial | 0.352 | [21, 24] |
| Fraction of misdemeanors that result in transitions to non-PWID, non-PWUD population via drug court | 0.005 | [29] |

**Table H. Transmission (Contact and Transfer) Parameters^[[9]](#footnote-10)^**

| **Parameter** | **Value** | **Source** |
| --- | --- | --- |
| **Drug Injection** |  |  |
| Number of risky injections per month (*N*) | 12 | [14, 73] |
| P(share per injection, casual) | 0.062 | Estimated [7] |
| P(share per injection, main) | 0.18 | Estimated [7] |
| **Sexual Contact** |  |  |
| N sex acts per month, average | 7 | [39] |
| NSP sharing multiplier | 0.55 | [26] |
| HIV aware *N* multiplier | 0.90 | [27] |
| SUDT *N* multiplier | 0.45 | [27] |
| **Condom Usage (probability per act)** |  | Estimated [6, 7, 19, 40] |
| **HIV uninfected/unaware** |  |  |
| **Female** |  |  |
| PWID with main partner | 0.44 |  |
| PWID with casual partner | 0.76 |  |
| PWUD with main partner | 0.19 |  |
| PWUD with casual partner | 0.57 |  |
| Non-drug user with main partner | 0.37 |  |
| Non-drug user with casual partner | 0.56 |  |
| **Heterosexual male** |  |  |
| PWID with main partner | 0.44 |  |
| PWID with casual partner | 0.76 |  |
| PWUD with main partner | 0.74 |  |
| PWUD with casual partner | 0.85 |  |
| Non-drug user with main partner | 0.63 |  |
| Non-drug user with casual partner | 0.72 |  |
| **MSM** |  |  |
| PWID with main partner | 0.44 |  |
| PWID with casual partner | 0.76 |  |
| PWUD with main partner | 0.51 |  |
| PWUD with casual partner | 0.64 |  |
| Non-drug user with main partner | 0.63 |  |
| Non-drug user with casual partner | 0.72 |  |
| **HIV aware** |  |  |
| **Female** |  |  |
| PWID with main partner | 0.58 |  |
| PWID with casual partner | 0.85 |  |
| PWUD with main partner | 0.25 |  |
| PWUD with casual partner | 0.74 |  |
| Non-drug user with main partner | 0.47 |  |
| Non-drug user with casual partner | 0.73 |  |
| **Heterosexual male** |  |  |
| PWID with main partner | 0.58 |  |
| PWID with casual partner | 0.85 |  |
| PWUD with main partner | 0.85 |  |
| PWUD with casual partner | 0.85 |  |
| Non-drug user with main partner | 0.82 |  |
| Non-drug user with casual partner | 0.85 |  |
| **MSM** |  |  |
| PWID with main partner | 0.58 |  |
| PWID with casual partner | 0.85 |  |
| PWUD with main partner | 0.66 |  |
| PWUD with casual partner | 0.83 |  |
| Non-drug user with main partner | 0.82 |  |
| Non-drug user with casual partner | 0.85 |  |

**Table I. Disease-Specific Transmission Parameters^[[10]](#footnote-11)^**

| **Parameter** | **Value** | **Source** |
| --- | --- | --- |
| **HIV** |  |  |
| **Transmission** |  | [6, 8, 41] |
| **Female to male** |  |  |
| VL>10 | 0.00001 |  |
| VL>40 | 0.00002 |  |
| VL>400 | 0.00008 |  |
| VL>1000 | 0.0001 |  |
| VL>10,000 | 0.0003 |  |
| VL>50,000 | 0.0007 |  |
| Acute HIV infection | 0.0055 |  |
| AIDS | 0.0027 |  |
| **Male to female** |  |  |
| VL>10 | 0.00003 |  |
| VL>40 | 0.00007 |  |
| VL>400 | 0.0002 |  |
| VL>1000 | 0.0003 |  |
| VL>10,000 | 0.0008 |  |
| VL>50,000 | 0.0016 |  |
| Acute HIV infection | 0.0112 |  |
| AIDS | 0.0059 |  |
| **Male to male** |  |  |
| VL>10 | 0.00007 |  |
| VL>40 | 0.0001 |  |
| VL>400 | 0.0004 |  |
| VL>1000 | 0.0006 |  |
| VL>10,000 | 0.0016 |  |
| VL>50,000 | 0.0035 |  |
| Acute HIV infection | 0.0215 |  |
| AIDS | 0.0114 |  |
| **Injection** |  |  |
| VL>10 | 0.00004 |  |
| VL>40 | 0.00009 |  |
| VL>400 | 0.0002 |  |
| VL>1000 | 0.0004 |  |
| VL>10,000 | 0.0011 |  |
| VL>50,000 | 0.0022 |  |
| Acute HIV infection | 0.0155 |  |
| AIDS | 0.0082 |  |
| Condom effectiveness | 0.9 | [27] |
| **HCV** |  |  |
| Transmission per injection | 0.075 | [14, 43] |
| Sexual transmission per partner per month | 0.00046 | [14, 42] |
| Injection damper, treatment | 0.5 | [14] |
| Sexual damper, treatment | 0.1 | [14] |
| Condom effectiveness | 0.79 | [14] |

##

**Table J. HIV Progression Parameters^[[11]](#footnote-12)^**

| **Parameter** | **Value** | **Source** |
| --- | --- | --- |
| Duration of acute phase (months) | 3 | [6] |
| **Progression in the absence of ART** |  |  |
| **CD4 decrease, normal distribution (mean, SD)** |  | [45] |
| VL < 500 | (1.67, 0.9) |  |
| VL < 2000 | (3.33, 1.7) |  |
| VL < 10000 | (4.08, 2.1) |  |
| VL < 40000 | (4.58, 2.4) |  |
| VL = 40000+ | (6.33, 3.2) |  |
| **Progression with ART** |  |  |
| CD4 rise on ART multiplier^[[12]](#footnote-13)^ | 75 | [44] |
| CD4 rise multiplier, > 40 years old | 0.80 | [44] |
| **Adherence multiplier** |  | [6, 46] |
| <30% adherent | 0 |  |
| 30-49% adherent | 0.20 |  |
| 50-69% adherent | 0.61 |  |
| 70-89% adherent | 0.82 |  |
| $\geq$90% adherent | 1.00 |  |
| VL decrease on ART, first 6 months | 0.92 | [44] |
| **Opportunistic infections (OIs)** |  | [44] |
| **Monthly risk given CD4 count** |  |  |
| 0-49 | 0.024 |  |
| 51-100 | 0.012 |  |
| 101-200 | 0.009 |  |
| 201-300 | 0.006 |  |
| 301-400 | 0.002 |  |
| 401-500 | 0.0006 |  |
| 500+ | 0 |  |
| CD4 decline with OI | 58.5 |  |

**Table K. HCV Progression Parameters^[[13]](#footnote-14)^**

| **Parameter** | **Value** | **Source** |
| --- | --- | --- |
| Duration of acute phase, months | 6 | [47] |
| **Monthly Fibrosis Progression** |  | [13] |
| **No HIV** |  |  |
| **Female** | 0.0017 |  |
| 18-19 | 0.0033 |  |
| 20-29 | 0.0054 |  |
| 30-49 | 0.0071 |  |
| 50-59 | 0.0141 |  |
| 60-69 | 0.0182 |  |
| 70-74 |  |  |
| **Male** | 0.0021 |  |
| 18-19 | 0.0042 |  |
| 20-29 | 0.0075 |  |
| 30-49 | 0.0104 |  |
| 50-59 | 0.0206 |  |
| 60-69 | 0.0287 |  |
| 70-74 | 0.0017 |  |
| **HIV, no ART** |  |  |
| **Female** |  |  |
| 18-19 | 0.0052 |  |
| 20-29 | 0.0103 |  |
| 30-49 | 0.0185 |  |
| 50-59 | 0.0256 |  |
| 60-69 | 0.0506 |  |
| 70-74 | 0.0701 |  |
| **Male** |  |  |
| 18-19 | 0.0041 |  |
| 20-29 | 0.0083 |  |
| 30-49 | 0.0134 |  |
| 50-59 | 0.0175 |  |
| 60-69 | 0.0347 |  |
| 70-74 | 0.0446 |  |
| **HIV, on ART** |  |  |
| **Female** |  |  |
| 18-19 | 0.0036 |  |
| 20-29 | 0.0071 |  |
| 30-49 | 0.0128 |  |
| 50-59 | 0.0178 |  |
| 60-69 | 0.0352 |  |
| 70-74 | 0.0490 |  |
| **Male** |  |  |
| 18-19 | 0.0029 |  |
| 20-29 | 0.0057 |  |
| 30-49 | 0.0093 |  |
| 50-59 | 0.0121 |  |
| 60-69 | 0.0241 |  |
| 70-74 | 0.0310 |  |
| **HCV Treatment** |  |  |
| **Probability of SVR** |  |  |
| HIV-, white, CC | 0.83 | [9] |
| HIV-, white, non-CC | 0.64 | [9] |
| HIV-, black, CC | 0.67 | [9] |
| HIV-, black, non-CC | 0.4 | [9] |
| Decrease in SVR, HIV+ | 0.89 | [14] |
| Decrease in SVR for F3, F4 | 0.80 | [9] |
| **Spontaneous clearing** |  | [9, 48] |
| Acute, HIV+ | 0.15 |  |
| Acute, HIV- | 0.25 |  |
| F0, HIV+ | 0.001 |  |
| F0, HIV- | 0.0006 |  |

**Table L. HIV and HCV Awareness and Treatment^[[14]](#footnote-15)^**

| **Parameter** | **Value** | **Source** |
| --- | --- | --- |
| **HCV treatment** |  |  |
| **Monthly probability of quitting** |  | Estimated [6, 46] |
| Adherence level 5 | 0.0085 |  |
| Adherence level 4 | 0.0270 |  |
| Adherence level 3 | 0.0365 |  |
| Adherence level 2 | 0.109 |  |
| Adherence level 1 | 0.125 |  |
| Treatment entry multiplier relative to ART, non-PWID, non-PWUD | 0.100 | Calibrated [11, 12] |
| Treatment entry multiplier relative to ART, PWID or PWUD | 0.025 | Calibrated [11, 12] |
| **HIV awareness (annual)** |  | Calibrated [6] |
| P(HIV test\|no NSP), PWID | 0.081 |  |
| P(HIV test\|NSP), PWID | 0.104 |  |
| P(HIV test), PWUD or non-drug user | 0.268 |  |
| **HIV treatment (monthly^[[15]](#footnote-16)^)** |  | Calibrated [6] |
| P(start ART\|no SUDT), PWID | 0.175 |  |
| P(start ART\|no SUDT), PWUD | 0.176 |  |
| P(start ART), non-drug user | 0.058 |  |
| P(start ART\| SUDT), PWID | 0.265 |  |
| P(start ART\| SUDT), PWUD | 0.244 |  |
| P(quit ART\|no SUDT), PWID | 0.0017 |  |
| P(quit ART\|no SUDT), PWUD | 0.0017 |  |
| P(quit ART), non-drug user | 0.0025 |  |
| P(quit ART\| SUDT), PWID | 0.0008 |  |
| P(quit ART\| SUDT), PWUD | 0.0008 |  |
| VL minimum when ART is ceased (log_10_ copies/ml) | 3 | [44] |
| Months since quitting ART before time on ART can reset | 4 | Calibrated |

**Table M. SUDT and NSP Program Parameters (monthly values)^[[16]](#footnote-17)^**

| **Parameter** | **Value** | **Source** |
| --- | --- | --- |
| **SUDT** |  |  |
| P(quit drug use via SUDT) | 0.003 | Calibrated [6] |
| P(stay waiting for SUDT) | 0.016 | Calibrated [51, 52] |
| P(get in for SUDT) | 0.638 | Calibrated [51, 52] |
| P(enter SUDT), PWUD | 0.018 | Calibrated [6] |
| P(enter SUDT\|no NSP), PWID | 0.018 | Calibrated [6] |
| P(enter SUDT\|NSP), PWID | 0.039 | Calibrated [6] |
| P(quit SUDT) | 0.028 | Calibrated [6] |
| **NSP** |  | Calibrated [6] |
| P(enter NSP),PWID | 0.049 |  |
| P(quit NSP), PWID | 0.029 |  |

**Table N. Diversion Program Parameters^[[17]](#footnote-18)^**

| **Parameter** | **Value** | **Source** |
| --- | --- | --- |
| Multiplier for criminal activity if connected to the diversion program (does not include effects of SUDT) | 0.58 | Estimated [74, 75] |
| Given a crime is commited, reduction in chance it is a felony charge if connected to the diversion program | 1 | Estimated [74, 75] |
| Diversion program QALY multiplier | 1 | Estimated |
| Diversion program annual cost (first three years) | $899 | [76] |
| Diversion program annual cost after start up | $532 | [76] |
| Multiplier for joining community programs if connected to the diversion program | 2 [1,3] | Control variable |
| Multiplier for quitting community programs if connected to the diversion program | 0.5 [1, 0.25] | Control variable |
| Fraction of misdemeanor arrests that result in entry to the diversion program | 0.25 [0.10, 0.75] | Control variable |

**Table O. Demographic Transition Parameters^[[18]](#footnote-19)^**

| **Parameter** | **Value** | **Source** |
| --- | --- | --- |
| **Drug-use transitions (probability per month)** |  |  |
| Non-drug user to PWID, ages $\leq$ 29 | 1.73E-05 | Calibrated [56] |
| Non-drug user to PWID, ages > 29 | 2.87E-06 | Calibrated [56] |
| PWUD to PWID | 0.000646 | Calibrated [57, 58] |
| Non-drug user to PWUD, ages $\leq$ 29 | 0.000442 | Calibrated [56] |
| Non-drug user to PWUD, ages > 29 | 7.17E-05 | Calibrated [56] |
| P(quit drug use via SUDT) | 0.0031 | [27] |

**Table P. Mortality Parameters**

| **Parameter** | **Value** | **Source** |
| --- | --- | --- |
| **Background^[[19]](#footnote-20)^** |  | [59] |
| **HCV** |  | [15] |
| **Hazard ratio, stages F0-F4** |  |  |
| **Female** | 1.05 |  |
| White | 1.11 |  |
| Black | 1.08 |  |
| Other |  |  |
| **Male** | 1.17 |  |
| White | 1.24 |  |
| Black | 1.21 |  |
| Other |  |  |
| Monthly probability of death, ESLD | 0.013 |  |
| **HIV** |  |  |
| **Monthly probability of death with an opportunistic infection** |  | [44, 45] |
| 0-50 | 0.0238 |  |
| 51-100 | 0.0119 |  |
| 101-200 | 0.0088 |  |
| 201-300 | 0.0058 |  |
| 301-400 | 0.0024 |  |
| 401-500 | 0.0006 |  |
| > 500 | 0 |  |
| **Additional HIV mortality** |  | [44] |
| **No ART, monthly probability** |  |  |
| 0-50 | 0.0173 |  |
| 51-100 | 0.0068 |  |
| 101-200 | 0.0054 |  |
| 201-300 | 0.0043 |  |
| 301-400 | 0.0036 |  |
| 401-500 | 0.0029 |  |
| > 500 | 0.0018 |  |
| **ART, monthly probability** |  |  |
| 0-50 | 0.0050 |  |
| 51-100 | 0.0028 |  |
| 101-200 | 0.0021 |  |
| 201-300 | 0.0015 |  |
| 301-400 | 0.0014 |  |
| 401-500 | 0.0008 |  |
| > 500 | 0.0005 |  |
| **Overdose deaths** |  |  |
| PWID, monthly probability | 0.00019 | [31, 36, 60-62] |
| PWUD, monthly probability | 0.00023 | [31, 36, 60, 61] |
| Multiplier from SUDT | 0.4 | [27, 32, 62] |
| PWID probability of post-incarceration overdose | 0.0095 | [33-36, 62] |

**Table Q. Costs and QALYs ^[[20]](#footnote-21)^**

| **Parameter** | **Value** | **Source** |
| --- | --- | --- |
| **QALY multipliers** |  |  |
| **Annual baseline** |  | [64] |
| **Female** |  |  |
| 18-19 | 0.91 |  |
| 20-29 | 0.90 |  |
| 30-39 | 0.89 |  |
| 40-49 | 0.86 |  |
| 50-59 | 0.83 |  |
| 60-74 | 0.81 |  |
| **Male** |  |  |
| 18-19 | 0.94 |  |
| 20-29 | 0.93 |  |
| 30-39 | 0.91 |  |
| 40-49 | 0.88 |  |
| 50-59 | 0.85 |  |
| 60-74 | 0.84 |  |
| **Drug use** |  |  |
| PWID | 0.90 | [27] |
| PWUD | 0.95 | [27] |
| **HIV** |  | [65] |
| CD4 >200 | 0.94 |  |
| CD4 101-200 | 0.87 |  |
| CD4 51-100 | 0.81 |  |
| CD4 0-50 | 0.79 |  |
| Opportunistic infection (replaces CD4 multiplier) | 0.60 |  |
| SUDT | 1.06 | [27] |
| NSP | 1.00 | [26] |
| **HCV** |  | [9] |
| F0/F1 | 0.98 |  |
| F2/F3 | 0.85 |  |
| F4 | 0.79 |  |
| ESLD | 0.72 |  |
| Monthly treatment decrement | -0.011 |  |
| SVR following F0/F1 | 1.00 |  |
| SVR following F2/F3 | 0.93 |  |
| SVR following F4 | 0.93 |  |
| Jail | 1.0 | Estimated |
| Prison | 1.0 | Estimated |
| **Monthly costs (2016 US $)** |  |  |
| **Background healthcare cost** |  | [27, 66] |
| Age < 60 | 335 |  |
| Age $\geq$60 | 714 |  |
| **Additional costs** |  |  |
| **HIV** |  |  |
| CD4 > 50 | 617 | [65] |
| CD4 0-50 | 1,036 | [65] |
| Opportunistic infection (in addition to CD4 cost) | 10,052 | [65] |
| ART | 1,250 | [27] |
| One-time cost of diagnosis | 500 | [27] |
| OAT | 540 | [27, 72] |
| NSP | 51 | [26] |
| **HCV** |  | [9] |
| F0/F1/F2/F3 | 125 |  |
| F4 | 375 |  |
| ESLD | 1,762 |  |
| Treatment | 8,500 |  |
| SVR following F0/F1/F2/F3 | 36 |  |
| SVR following F4 | 73 |  |
| **Incarceration** |  |  |
| One-time law enforcement cost | 1,100 | [21] |
| One-time misdemeanor cost | 273 | [21] |
| One-time felony cost | 9,740 | [21] |
| One-time jail booking fee | 121 | [21] |
| Jail (weekly) | 679 | [21] |
| Prison | 3,188 | [21, 67] |

**References**

1. Bernard CL, Brandeau ML. Structural sensitivity in HIV modeling: a case study of vaccination. Infect Dis Model. 2017;2(4):399-411.

2. Buskin S, Hanrahan M, Jaenicke T. HIV/AIDS epidemiology report 2015 (volume 84) Seattle, WA: HIV/AIDS Epidemiology Unit, Public Health – Seattle & King County and the Infectious Disease Assessment Unit, Washington State Department of Health; 2016. Available from: <http://www.kingcounty.gov/healthservices/health/communicable/hiv/epi/~/media/health/publichealth/documents/hiv/2015EpiReport.ashx>.

3. Valdiserri RO, Koh HK. Breaking the silence on viral hepatitis. Ann Intern Med. 2014;161(2):147-8.

4. Hellard M, Rolls DA, Sacks-Davis R, Robins G, Pattison P, Higgs P, et al. The impact of injecting networks on hepatitis C transmission and treatment in people who inject drugs. Hepatology. 2014;60(6):1861-70.

5. Buskin S, Hanrahan M, Jaenicke T. HIV/AIDS epidemiology report, first half 2013 (volume 82) Seattle, WA: HIV/AIDS Epidemiology Unit, Public Health – Seattle & King County and the Infectious Disease Assessment Unit, Washington State Department of Health; 2013. Available from: <http://www.kingcounty.gov/healthservices/health/communicable/hiv/epi/~/media/health/publichealth/documents/hiv/1stHalf2013EpiReport.ashx>.

6. Monteiro JF, Escudero DJ, Weinreb C, Flanigan T, Galea S, Friedman SR, et al. Understanding the effects of different HIV transmission models in individual-based microsimulation of HIV epidemic dynamics in people who inject drugs. Epidemiol Infect. 2016;144(8):1683-700.

7. Broz D, Wejnert C, Pham HT, DiNenno E, Heffelfinger JD, Cribbin M, et al. HIV infection and risk, prevention, and testing behaviors among injecting drug users -- National HIV Behavioral Surveillance System, 20 U.S. cities, 2009. Morbidity and mortality weekly report Surveillance summaries. 2014;63(6):1-51.

8. Bendavid E, Brandeau ML, Wood R, Owens DK. Comparative effectiveness of HIV testing and treatment in highly endemic regions. Arch Intern Med. 2010;170(15):1347-54.

9. Liu S, Watcha D, Holodniy M, Goldhaber-Fiebert JD. Sofosbuvir-based treatment regimens for chronic, genotype 1 hepatitis C virus infection in U.S. incarcerated populations: a cost-effectiveness analysis. Ann Intern Med. 2014;161(8):546-53.

10. University of Washington. Hepatitis C online 2017 [cited 2018 Apr 3]. Available from: <http://www.hepatitisc.uw.edu/>

11. Mehta SH, Genberg BL, Astemborski J, Kavasery R, Kirk GD, Vlahov D, et al. Limited uptake of hepatitis C treatment among injection drug users. J Community Health. 2008;33(3):126-33.

12. Grebely J, Genoway KA, Raffa JD, Dhadwal G, Rajan T, Showler G, et al. Barriers associated with the treatment of hepatitis C virus infection among illicit drug users. Drug Alcohol Depend. 2008;93(1-2):141-7.

13. Salomon JA, Weinstein MC, Hammitt JK, Goldie SJ. Empirically calibrated model of hepatitis C virus infection in the United States. Am J Epidemiol. 2002;156(8):761-73.

14. Cipriano LE, Zaric GS, Holodniy M, Bendavid E, Owens DK, Brandeau ML. Cost effectiveness of screening strategies for early identification of HIV and HCV infection in injection drug users. PLoS ONE. 2012;7(9):e45176.

15. Liu S, Cipriano LE, Holodniy M, Owens DK, Goldhaber-Fiebert JD. New protease inhibitors for the treatment of chronic hepatitis C: a cost-effectiveness analysis. Ann Intern Med. 2012;156(4):279-90.

16. Hamilton DT, Handcock MS, Morris M. Degree distributions in sexual networks: a framework for evaluating evidence. Sex Transm Dis. 2008;35(1):30-40.

17. Marshall BDL, Wood E. Towards a comprehensive approach to HIV prevention for people who use drugs. J Acquir Immune Defic Syndr. 2010;55 Suppl 1:S23-S6.

18. National Center for Health Statistics. National survey of family growth, 2013-2015 2016 [cited 2018 Jan 20]. Available from: <https://www.cdc.gov/nchs/nsfg/index.htm>.

19. Kapadia F, Latka MH, Hudson SM, Golub ET, Campbell JV, Bailey S, et al. Correlates of consistent condom use with main partners by partnership patterns among young adult male injection drug users from five US cities. Drug Alcohol Depend. 2007;91 Suppl 1:S56-S63.

20. The National Center on Addiction and Substance Abuse at Columbia University. Behind Bars II: Substance Abuse and America’s Prison Population. New York: Columbia University, 2010.

21. Berk Consulting. Analysis of Statewide Adult Correctional Needs and Costs. Final Report Submitted to the Office of Financial Management, November 6, 2014. Washington State. 2014 [cited 2018 Jun 29]. Available from: <http://www.ofm.wa.gov/reports/Correctional_Needs_and_Costs_Study2014.pdf>.

22. Washington State Department of Corrections. Average daily population of incarcerated individuals, fiscal year 2017 2017 [cited 2018 Jun 29]. Available from: <http://www.doc.wa.gov/docs/publications/400-RE002.pdf>.

23. Washington State Office of Financial Management. Prison inmate population Seattle, Washington: The State of Washington; 2017 [updated January 9, 2017; cited 2018 Jun 29]. Available from: <http://www.ofm.wa.gov/trends/budget/fig408.asp>.

24. Washington State Department of Corrections. Number of prison admissions by county of admission. August 2015. 2015 [cited 2018 Jun 29]. Available from: <http://www.doc.wa.gov/information/data/docs/admissions-releases-by-county.pdf>.

25. King County Department of Adult and Juvenile Detention. Detention and alternatives report 2015 [cited 2018 Jun 29]. Available from: <http://www.kingcounty.gov/~/media/courts/detention/documents/KC_DAR_12_2015.ashx?la=en>.

26. Bernard CL, Owens DK, Goldhaber-Fiebert JD, Brandeau ML. Estimation of the cost-effectiveness of HIV prevention portfolios for people who inject drugs in the United States: a model-based analysis. PLoS Med. 2017;14(5):e1002312.

27. Bernard CL, Brandeau ML, Owens DK, Humphreys K, Bendavid E, Weyant C, et al. Cost-effectiveness of HIV preexposure prophylaxis for people who inject drugs in the United States. Ann Intern Med. 2016;165(1):10-9.

28. Drake EK, Aos S, Miller MG. Evidence-based public policy options to reduce crime and criminal justice costs: implications in Washington State. Victims & Offenders. 2009;4(2):170-96.

29. King County Washington. King County Adult Drug Diversion Court 2016 [cited 2018 Jun 29]. Available from: <http://www.kingcounty.gov/courts/clerk/drug-court.aspx>.

30. King County Department of Adult and Juvenile Detention. Average daily population by housing location and demographics. December 2015. 2016 [cited 2018 Jun 29]. Available from: <http://www.kingcounty.gov/~/media/courts/detention/documents/KC_DAR_Monthly_Breakouts_CY2015.ashx?la=en>

31. Lee JD, McDonald R, Grossman E, McNeely J, Laska E, Rotrosen J, et al. Opioid treatment at release from jail using extended-release naltrexone: a pilot proof-of-concept randomized effectiveness trial. Addiction. 2015;110(6):1008-14.

32. Larney S, Gisev N, Farrell M, Dobbins T, Burns L, Gibson A, et al. Opioid substitution therapy as a strategy to reduce deaths in prison: retrospective cohort study. BMJ Open. 2014;4(4):e004666.

33. Maryland Department of Health and Mental Hygiene. Risk of overdose death following release from prison or jail 2014 [cited 2018 Jun 29]. Available from: <https://bha.health.maryland.gov/OVERDOSE_PREVENTION/Documents/corrections%20brief_V3.pdf>.

34. Rich JD, McKenzie M, Larney S, Wong JB, Tran L, Clarke J, et al. Methadone continuation versus forced withdrawal on incarceration in a combined US prison and jail: a randomised, open-label trial. Lancet. 2015;386(9991):350-9.

35. Degenhardt L, Larney S, Kimber J, Gisev N, Farrell M, Dobbins T, et al. The impact of opioid substitution therapy on mortality post-release from prison: retrospective data linkage study. Addiction. 2014;109(8):1306-17.

36. Aleccia J. Heroin deaths drop in King County, but addiction still a serious problem. Seattle Times. 2016 July 19.

37. Long EF, Brandeau ML, Owens DK. The cost effectiveness and population outcomes of expanded HIV screening and antiretroviral treatment in the United States. Ann Inter Med. 2010;153(12):778-89.

38. Long EF, Owens DK. The cost-effectiveness of a modestly effective HIV vaccine in the United States. Vaccine. 2011;29(36):6113-24.

39. Vickerman P, Watts C. The impact of an HIV prevention intervention for injecting drug users in Svetlogorsk, Belarus: model predictions. Int J Drug Policy. 2002;13(3):149-64.

40. Sionean C, Le BC, Hageman K, Oster AM, Wejnert C, Hess KL, et al. HIV risk, prevention, and testing behaviors among heterosexuals at increased risk for HIV infection -- National HIV Behavioral Surveillance System, 21 U.S. Cities, 2010. Morbidity and mortality weekly report Surveillance summaries. 2014;63(14):1-39.

41. Public Health Agency of Canada. HIV transmission risk: A summary of the evidence 2012 [cited 2018 Jun 29]. Available from: <http://www.catie.ca/sites/default/files/HIV-TRANSMISSION-RISK-EN.pdf>.

42. Terrault NA. Sexual activity as a risk factor for hepatitis C. Hepatology. 2002;36 Suppl 1(5):S99-S105.

43. Vickerman P, Hickman M, Judd A. Modelling the impact on Hepatitis C transmission of reducing syringe sharing: London case study. Int J Epidemiol. 2007;36(2):396-405.

44. Zhong H, Arjmand IK, Brandeau ML, Bendavid E. Clinical outcomes and cost-effectiveness of treating depression in HIV-infected populations in sub-Saharan Africa: a model-based analysis. AIDS Care. 2020; Jan 20:1-7, Epub ahead of print.

45. Bendavid E, Young SD, Katzenstein DA, Bayoumi AM, Sanders GD, Owens DK. Cost-effectiveness of HIV monitoring strategies in resource-limited settings – a Southern African analysis. Arch Intern Med. 2008;168(17):1910-8.

46. Chen NE, Meyer JP, Avery AK, Draine J, Flanigan TP, Lincoln T, et al. Adherence to HIV treatment and care among previously homeless jail detainees. AIDS Behav. 2013;17(8):2654-66.

47. Chen SL, Morgan TR. The natural history of hepatitis C virus (HCV) infection. Int J Med Sci. 2006;3(2):47-52.

48. Scott JA, Chew KW. Treatment optimization for HIV/HCV co-infected patients. Ther Adv Infect Dis. 2017;4(1):18-36.

49. Clifasefi SL, Collins SE. LEAD Program Evaluation: Describing LEAD Case Management in Participants’ Own Words: Harm Reduction Research and Treatment Lab at the University of Washington Harborview Medical Center; 2016 [cited 2017]. Available from: <http://leadkingcounty.org/lead-evaluation/>.

50. Rich JD, Chandler R, Williams BA, Dumont D, Wang EA, Taxman FS, et al. How health care reform can transform the health of criminal justice-involved individuals. Health Affairs (Millwood). 2014;33(3):462-7.

51. Chun J, Guydish JR, Silber E, Gleghorn A. Drug treatment outcomes for persons on waiting lists. Am J Drug Alcohol Abuse. 2008;34(5):526-33.

52. Redko C, Rapp RC, Carlson RG. Waiting time as a barrier to treatment entry: perceptions of substance users. J Drug Issues. 2006;36(4):831-52.

53. US Census Bureau. Population demographics for King County, Washington in 2016 and 2017 2017 [cited 2018 Jun 29]. Available from: <https://suburbanstats.org/population/washington/how-many-people-live-in-king-county>

54. Vance-Sherman A. King County profile Olympia, WA: Washington State Employment Security Department; 2015 [cited 2018 Jun 1]. Available from: <https://fortress.wa.gov/esd/employmentdata/reports-publications/regional-reports/county-profiles/king-county-profile>.

55. Seattle and King County Public Health Department. Facts about HIV/AIDS in incarcerated people 2009 [cited 2018 Jun 15]. Available from: <http://www.kingcounty.gov/healthservices/health/communicable/hiv/epi/~/media/health/publichealth/documents/hiv/HIVIncarcerated.ashx>.

56. Kingston S, Banta-Green C. Results from the 2015 Washington State Drug Injector Health Survey Seattle, Washington: University of Washington; 2016 [updated Feb 2016; cited 2018 Jun 29]. Available from: <http://adai.uw.edu/pubs/infobriefs/2015DrugInjectorHealthSurvey.pdf>.

57. van Ameijden EJ, van den Hoek JA, Hartgers C, Coutinho RA. Risk factors for the transition from noninjection to injection drug use and accompanying AIDS risk behavior in a cohort of drug users. Am J Epidemiol. 1994;139(12):1153-63.

58. Fuller CM, Vlahov D, Ompad DC, Shah N, Arria A, Strathdee SA. High-risk behaviors associated with transition from illicit non-injection to injection drug use among adolescent and young adult drug users: a case-control study. Drug Alcohol Depend. 2002;66(2):189-98.

59. Arias E. United States life tables, 2010. Natl Vital Stat Rep. 2014;63(7):1-63.

60. National Institute on Drug Abuse. Overdose death rates, revised 2017 2017 [cited 2018 Jun 29]. Available from: <https://www.drugabuse.gov/related-topics/trends-statistics/overdose-death-rates>.

61. Bird SM, Parmar MK, Strang J. Take-home naloxone to prevent fatalities from opiate-overdose: protocol for Scotland's public health policy evaluation, and a new measure to assess impact. Drugs (Abingdon Engl). 2015;22(1):66-76.

62. Larney S, Toson B, Burns L, Dolan K. Effect of prison-based opioid substitution treatment and post-release retention in treatment on risk of re-incarceration. Addiction. 2012;107(2):372-80.

63. Goldhaber-Fiebert JD, Stout NK, Ortendahl J, Kuntz KM, Goldie SJ, Salomon JA. Modeling human papillomavirus and cervical cancer in the United States for analyses of screening and vaccination. Popul Health Metr. 2007;5:11.

64. Nyman JA, Barleen NA, Dowd BE, Russell DW, Coons SJ, Sullivan PW. Quality-of-life weights for the US population: self-reported health status and priority health conditions, by demographic characteristics. Med Care. 2007;45(7):618-28.

65. Freedberg KA, Scharfstein JA, Seage 3rd GR, Losina E, Weinstein MC, Craven DE, et al. The cost-effectiveness of preventing AIDS-related opportunistic infections. JAMA. 1998;279(2):130-6.

66. Meara E, White C, Cutler DM. Trends in medical spending by age, 1963-2000. Health Affairs (Millwood). 2004;23(4):176-83.

67. Washington State Department of Corrections. FY2016 cost per incarcerated individual per day. 2017 [cited 2018 Mar 15]. Available from: <http://doc.wa.gov/docs/publications/reports/200-AR001.pdf>.

68. Gold M. Panel on Cost-Effectiveness in Health and Medicine. Med Care. 1996;34 Suppl(12 ):DS197- DS9.

69. Weinstein MC, O'Brien B, Hornberger J, Jackson J, Johannesson M, McCabe C, et al. Principles of good practice for decision analytic modeling in health-care evaluation: Report of the ISPOR Task Force on Good Research Practices--Modeling Studies. Value Health. 2003;6(1):9-17.

70. Suen SC, Bendavid E, Goldhaber-Fiebert JD. Cost-effectiveness of improvements in diagnosis and treatment accessibility for tuberculosis control in India. Int J Tuberc Lung Dis. 2014;19(9):1115-24.

71. Washington State Department of Corrections. Washington State prisons. March 2016. 2016 [cited 2018 Jun 29]. Available from: <http://www.doc.wa.gov/facilities/prison/>.

72. Evergreen Treatment Services. Prospective patients 2017 [cited 2018 Jun 29]. Available from: <https://www.evergreentx.org/patients/prospective-patients/>.

73. Leri F, Stewart J, Tremblay A, Bruneau J. Heroin and cocaine co-use in a group of injection drug users in Montréal. J Psychiatry Neurosci. 2004;29(1):40-7.

74. Collins SE, Lonczak HS, Clifasefi SL. LEAD Program Evaluation: Recidivism Report: Harm Reduction Research and Treatment Lab at the University of Washington Harborview Medical Center; 2015 [cited 2017]. Available from: <http://leadkingcounty.org/lead-evaluation/>.

75. Collins SE, Lonczak HS, Clifasefi SL. Seattle's Law Enforcement Assisted Diversion (LEAD): program effects on recidivism outcomes. Eval Program Plann. 2017;64:49-56.

76. Collins SE, Lonczak HS, Clifasefi SL. LEAD Program Evaluation: Criminal Justice and Legal System Utilization and Associated Costs: Harm Reduction Research and Treatment Lab at the University of Washington Harborview Medical Center; 2015 [cited 2017]. Available from: <http://leadkingcounty.org/lead-evaluation/>.

1. MSM = men who have sex with men. PWID = people who inject drugs. PWUD = people who use but do not inject drugs. [↑](#footnote-ref-2)
2. ART = antiretroviral therapy. MSM = men who have sex with men. PWID = people who inject drugs. PWUD = people who abuse but do not inject drugs. [↑](#footnote-ref-3)
3. A PWUD was identified in the following way. First, it was determined if the individual was a PWID or non-PWID. If non-PWID, it was then determined if the individual was a PWUD or not. [↑](#footnote-ref-4)
4. CC-type and non-CC-type refers to variants of the human IL28B gene. G1/2/3/4 genotype refer to variants of the HCV infection. HCV = hepatitis C virus. PWID = people who inject drugs. PWUD = people who abuse but do not inject drugs. [↑](#footnote-ref-5)
5. MSM = men who have sex with men. PWID = people who inject drugs. PWUD = people who abuse but do not inject drugs. [↑](#footnote-ref-6)
6. PWID = people who inject drugs. PWUD = people who abuse but do not inject drugs. [↑](#footnote-ref-7)
7. ART = antiretroviral therapy. NSP = needle/syringe exchange program. SUDT = treatment for substance use disorder. [↑](#footnote-ref-8)
8. PWID = people who inject drugs. PWUD = people who abuse but do not inject drugs. SUDT = treatment for substance use disorder. [↑](#footnote-ref-9)
9. MSM = men who have sex with men. NSP = needle/syringe exchange program. PWID = people who inject drugs. PWUD = people who abuse but do not inject drugs. SUDT = treatment for substance use disorder. [↑](#footnote-ref-10)
10. HCV = hepatitis C virus. VL = viral load. [↑](#footnote-ref-11)
11. ART = antiretroviral therapy. OI = opportunistic infection. VL = viral load. [↑](#footnote-ref-12)
12. The underlying CD4 rise is calculated as *log*(*m+2*)*/log*(*m+*1), where *m* is months on ART. [↑](#footnote-ref-13)
13. ART = antiretroviral therapy. CC-type and non-CC-type refers to variants of the human IL28B gene. HCV = hepatitis C virus. SVR = sustained virologic response. [↑](#footnote-ref-14)
14. ART = antiretroviral therapy. HCV = hepatitis C virus. PWID = people who inject drugs. PWUD = people who abuse but do not inject drugs. SUDT = treatment for substance use disorder. [↑](#footnote-ref-15)
15. Note, entrance rates are higher for drug users to counbalance the effects of frequent incarceration, which, at least temporarily, disconnects participants. [↑](#footnote-ref-16)
16. NSP = needle/syringe exchange program. PWID = people who inject drugs. PWUD = people who abuse but do not inject drugs. SUDT = treatment for substance use disorder. [↑](#footnote-ref-17)
17. SUDT = treatment for substance use disorder. QALY = quality-adjusted life year. [↑](#footnote-ref-18)
18. PWID = people who inject drugs. PWUD = people who abuse but do not inject drugs. SUDT = treatment for substance use disorder. [↑](#footnote-ref-19)
19. Background mortality rates by age, sex, and race are not reproduced here.

    ESLD = end stage liver disease. HCV = hepatitis C virus. PWID = people who inject drugs. PWUD = people who abuse but do not inject drugs. SUDT = treatment for substance use disorder. ART = antiretroviral therapy. [↑](#footnote-ref-20)
20. ART = antiretroviral therapy. ESLD = end stage liver disease. HCV = hepatitis C virus. MSM = men who have sex with men. NSP = needle/syringe exchange program. PWID = people who inject drugs. PWUD = people who abuse but do not inject drugs. QALY = quality-adjusted life year. SUDT = treatment for substance use disorder. SVR = sustained virologic response. [↑](#footnote-ref-21)
